# Supplementary material for: Theory of branching morphogenesis by local interactions and global guidance
Source: Nat Commun. 2021 Nov 24;12:6830. doi: 10.1038/s41467-021-27135-5 (PMC8613190; doi:10.1038/s41467-021-27135-5)
Supplement: Supplementary file 1 — Supplementary Information [file 41467_2021_27135_MOESM1_ESM.pdf]

Supplementary Information - Theory of branching morphogenesis by local interactions and global guidance

Contents

1 Supplementary Note 1: Derivation of the continuum model **2**
1.1 Alignment angle as a random variable 2
1.2 Fokker-Planck equation with non-local jumps 4
1.3 Fokker-Planck equation for the angle difference ψ 5

2 Supplementary Note 2: Simulation details **7**
2.1 Branching and annihilating random walks (BARWs) 7
2.2 BARWs with external guidance and/or self-avoidance 10
2.3 Estimating the average opening angle 13
2.4 Simulation results for BARWs with self-avoidance 13
2.5 Simulation of BARWs with external guidance *via* tip displacement 16
2.6 Simulation of BARWs with external guidance in three-dimensions 16
2.7 Parameter values used in the simulations 20

3 Supplementary Note 3: Experimental model system and methods **20**
3.1 Coarse-grained reconstruction of neuronal filaments 20
3.2 Analysis of experimental data 24

In this Supplementary Note, we provide additional details on the model derivation, on the numerical simulations of branching with repulsion and external guidance, as well as on the data collection, analysis and fitting to the model.

1 Supplementary Note 1: Derivation of the continuum model

In the continuum description, we concentrate on incorporating external guidance into a model of branching and annihilating random walks, in order to make simple predictions for the orientation of branches/tips during branching morphogenesis.

1.1 Alignment angle as a random variable

The growth of a branching tissue with active tips in an external potential can be in principle modelled in the framework of a *biased and persistent* random walk in two dimensions. However, in such a framework, a full expression for the time evolution of the probability density $p(\mathbf{r}, t)$ for an active particle to be at location $\mathbf{r}$ at time t in general cannot be obtained in a closed form [1], and will involve several bias and reorientation terms due to different frequencies arising from elongation vs. branching events. We therefore sought to reformulate the problem in a simpler framework for a single random variable. After realizing that if we focus on the *alignment* of branch segments with an external field, rather than their exact position and polarity, we could restate the problem as finding how much the local angles of the segments diverge from the local direction of the external field. To avoid confusion, we note that by *branch segments* we refer to the elementary vectors of a fixed size ℓ, corresponding to the discrete steps taken by active tips, that are linked together consecutively to define the branches of a tree.

Angular alignment of a branch segment is quantified by different angles for the two cases of external fields discussed in the main text: (i) For a horizontally oriented, axial external gradient (directed towards the positive x direction), the *local angle* φ of a branch segment with respect to the horizontal axis determines its alignment along the field. (ii) For a radial external field emerging from a central point of origin (which is relevant for the experimental geometry of the zebrafish fin), however, the angular alignment will be given by the *angle difference* ψ:

$$\psi \equiv \varphi - \theta \,, \tag{S1}$$

where θ denotes the angle of the active tip coordinates with respect to the origin of the external field. Supplementary Fig. 1(A) illustrates the alignment angles for the two different external potentials. Note that, the axial potential is a limiting case of a radial field where the origin of the external field is located at $x \to -\infty$. This choice then corresponds to setting $\theta = 0$ where the alignment angle ψ becomes equal to the local angle φ. Because of its generality, we will derive our model for the radial case using the angle difference ψ as the alignment angle in the following. Expressions for the axial case can then be obtained simply by setting $\theta = 0$.

1.1.1 Effect of an external field on the angle difference ψ

We now consider an external field that will influence the directionality of growing tips. At each step, the angle of a given active tip will be modified both stochastically via the randomness associated to elongation direction and branching, but also deterministically via guidance from the external field. For a branching network invading a circular region (in two-dimensions) we can define a radial potential that will displace the tips towards the point P with coordinates given by the vector $\mathbf{r} + f_c\,\mathbf{p}_c$, where f_c determines the strength of the external field, $\mathbf{r}$ is the distance vector of length $r\,\ell$ between the active

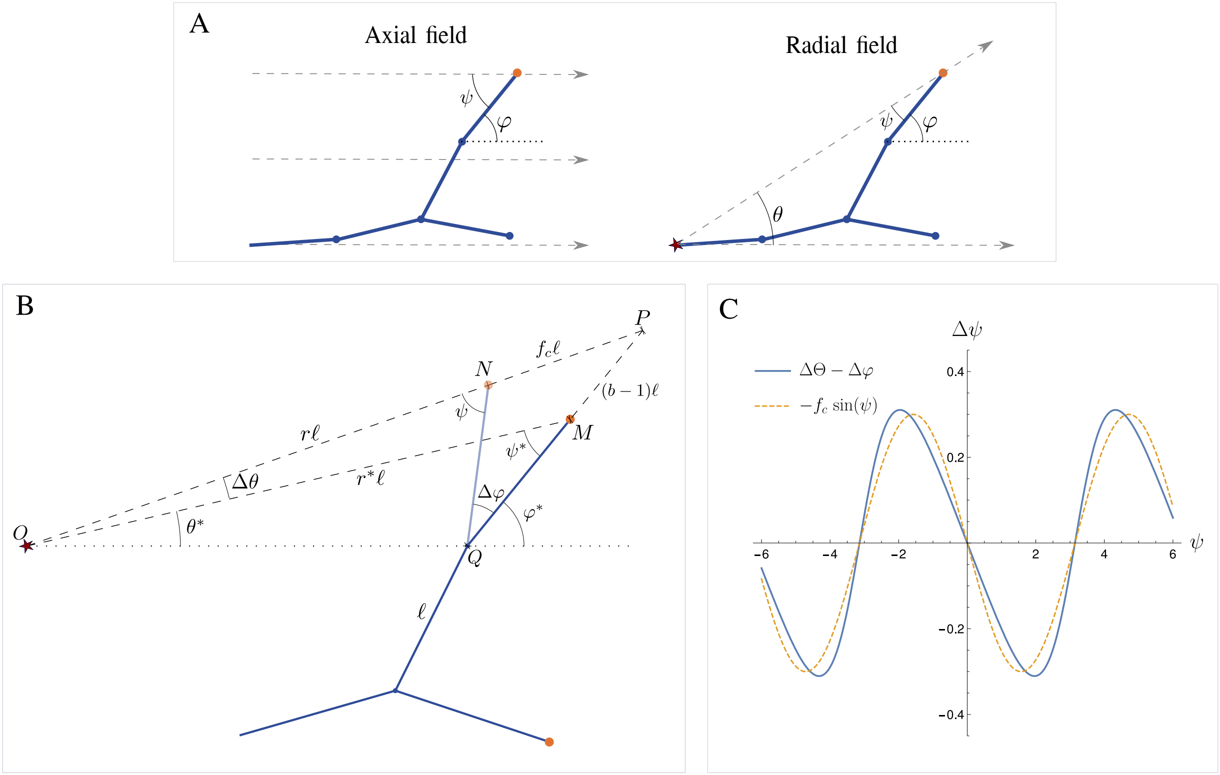

Supplementary Figure 1: **Definition and schematic of the variables used in the continuum model.** (A) Schematic of branch segments in an axial (left) or radial (right) external field (dashed arrows) in two-dimensions (with x, y coordinates as shown) highlighting the main variables used in the analysis: In a radial field (right), for every branch segment with an active tip (orange node) one can define the *local angle* φ that it makes with the horizontal axis (dotted lines), its *angle* θ *to origin* (star symbol), and the *angle difference* $\psi \equiv \varphi - \theta$. Accordingly, the alignment of a branch segment with the field is determined by the angle difference ψ, whereas for an axial field (lefty), the origin of the external field is located at $x \to -\infty$ (corresponding to $\theta = 0$), and thus the alignment will be determined by the local angle φ only ($\psi = \varphi$). Blue solid lines represent (static) branch segments of size ℓ generated by tip elongation (blue circles indicate inactive tips after an annihilation event due to proximity to other branches) (B) Displacement of an active tip due to a radial field. The external field acts along the radial distance $r\ell$ of the active tip to the origin of the potential (labelled by star) with a strength determined by the coupling parameter f_c. The branch segment is then aligned towards the extended point P but preserves its length ℓ. The angle difference after displacement $\psi^* = \varphi^* - \theta^*$ is then given by Eqs.(S2-S4), and can be approximated as a function of the field strength f_c and of its value ψ before displacement only, see Eq.(S5). (C) Plot of the changes in the angle difference $\Delta\psi$ after the displacement due to the external field f_c. The exact expression given by Eqs.(S2-S4) is compared with the approximation $\Delta\psi = -f_c \sin(\psi)$ for a value of $f_c = 0.3$, showing good overall agreement. For smaller values of f_c that are explored in the implementation of tip displacement-based simulations, the two expressions converge onto each other.

tip and the field origin, and $\mathbf{p}_c$ is a vector of length ℓ parallel to $\mathbf{r}$. We note that we discretise the problem by having tip elongation by a small length $\ell = 1$ (relative to the overall size of the network) without loss of generality. After such a displacement by the external field, the angle difference is given by

$$\psi^* = \psi + \Delta\theta - \Delta\varphi = \varphi^* - \theta^* \,, \tag{S2}$$

where $\Delta\varphi \equiv \varphi - \varphi^*$ and $\Delta\theta \equiv \theta - \theta^*$ denote respectively the changes in the local angle and in the angle to origin values. To express the new angle ψ^* in terms of ψ and the external field strength f_c, we deduce the following trigonometric relations from simple geometric arguments, see Supplementary

Fig. 1(B):

$$\Delta\varphi = \arcsin\left(\frac{f_c}{b}\sin(\psi)\right) , \tag{S3}$$

and

$$\Delta\theta = \arcsin\left(\frac{b-1}{r^* b}\sin(\psi)\right) , \tag{S4}$$

where r^* represents the distance of the active tip to the field origin *after* the displacement by the external field, and b determines the distance $b\,\ell$ between the point P and the branch node Q, see Supplementary Fig. 1(B). Note that, to obtain Eq. (S4) we have used the sine rule twice for the triangles OMP and PQN. For a wide range of values of f_c, variations in the local angle $\Delta\varphi$ strongly dominate over the term $\Delta\theta$, such that the latter can be neglected. Furthermore, for the weak field case that we investigate here, *i.e.*, for low values of f_c, the term $\Delta\varphi$ can be simply expressed by $f_c \sin(\psi)$ because the length scale b takes values close to 1. The change in the angle difference after displacement can then be approximated by the following expression:

$$\Delta\psi \equiv \psi^* - \psi = -f_c \sin(\psi) , \tag{S5}$$

which is a function of f_c and ψ only. Supplementary Fig. 1(C) provides a comparison between this approximation and the exact relation determined by Eqs.(S2-S4). This dependency on the external field f_c reflects a sinusoidal reorientation model for the angle difference ψ which was previously demonstrated for the gyrotaxis of spherical microswimmers [2], for cyanobacterial circadian oscillators [3] and for dipoles in a constant external field [4].

1.2 Fokker-Planck equation with non-local jumps

In simulations of branching and annihilating random walks (BARWs), elongation events lead to a small rotational diffusion of the branch segments, whereas branching events lead to large, abrupt changes in the local angle values of the active tips, as illustrated in Supplementary Fig. 2(A). These two different sources of noises are described by an *a priori* probability distribution $\lambda(\varphi - \varphi')$ for the difference in the local angles before φ' and after φ a jump. The external field then subsequently acts to modify these jump probabilities. This simulation setup can thus be described by separating the jumps arising from elongation and branching events from the bias arising through the external potential. Because of the large, non-local jumps in the local angle values φ, the standard method of reaching the continuum limit by expanding around small $\Delta\varphi$ cannot be performed. We will therefore follow a generalized method developed for Lévy flights in Refs.[5, 6] to derive the Fokker-Planck equation.

We first derive the central result of Ref.[6] for generic non-local jumps in one spatial dimension (represented by the continuous variable x) and then apply it to the angle difference ψ. We start with the continuum version of the difference equation for the probability distribution $P(x, t + \Delta t)$ after a jump from site x' to x within the time interval Δt:

$$P(x, t + \Delta t) = \int dx' \Lambda(x, x') P(x', t) , \tag{S6}$$

where the transfer kernel

$$\Lambda(x, x') \equiv 2\,\lambda(x - x')[A(x')H(x - x') + B(x')H(x' - x)] \tag{S7}$$

encodes both the distance between the jumps $x - x'$ via the corresponding probability density function $\lambda(x - x')$, and the spatial dependency of the transition probabilities $A(x)$ and $B(x)$. $H(z)$ denotes the Heaviside step function defined as $H(z) = 1$ for $z \geq 0$ and $H(z) = 0$ otherwise. We furthermore assume symmetric jump distance distributions of the form $\lambda(x - x') = \lambda(x' - x)$, *i.e.*, the probability

for different jump sizes only depends on the size of the jump and not on the direction. The directional biases are governed by the transition probabilities $A(x)$ and $B(x)$. The normalization for the transfer kernel is defined by integrating Eq.(S7) over the jump differences $z \equiv x - x'$:

$$2\left(A(x')\int_0^\infty dz\,\lambda(z) + B(x')\int_{-\infty}^0 dz\,\lambda(z)\right) = A(x') + B(x') = 1\,, \tag{S8}$$

where we used the symmetry of $\lambda(z)$ together with its normalization $\int_{-\infty}^\infty dz\,\lambda(z) = 1$. We now multiply Eq.(S6) by e^{ikx} and integrate over dx to obtain

$$\begin{aligned}\int_{-\infty}^\infty dx\, e^{ikx} P(x, t+\Delta t) = 2\int_{-\infty}^\infty dx' P(x',t)A(x')\int_{-\infty}^\infty dx\, e^{ikx}\lambda(x-x')H(x-x') \\ +2\int_{-\infty}^\infty dx' P(x',t)B(x')\int_{-\infty}^\infty dx\, e^{ikx}\lambda(x-x')H(x'-x)\,.\end{aligned} \tag{S9}$$

Substituting $z = x - x'$ in the integrals over x and using the Euler identity we get:

$$\begin{aligned}P(k, t+\Delta t) = \int_{-\infty}^\infty dx' P(x',t)A(x')e^{ikx'}\lambda_+(k) \\ + \int_{-\infty}^\infty dx' P(x',t)B(x')e^{ikx'}\lambda_-(k)\,,\end{aligned} \tag{S10}$$

where we introduced the Fourier transform of $P(x,t)$ and define

$$\begin{aligned}\lambda_+(k) \equiv & 2\left(\int_0^\infty dz\cos(kz)\lambda(z) + i\int_0^\infty dz\sin(kz)\lambda(z)\right)\,, \\ \lambda_-(k) \equiv & 2\left(\int_{-\infty}^0 dz\cos(kz)\lambda(z) + i\int_{-\infty}^0 dz\sin(kz)\lambda(z)\right)\,.\end{aligned} \tag{S11}$$

By switching the integration bounds for $\lambda_-(k)$ and using symmetry properties of sine and cosine functions, we can express Eq.(S10) as follows

$$\begin{aligned}P(k, t+\Delta t) = & 2\int_0^\infty dz\cos(kz)\lambda(z)\int_{-\infty}^\infty dx' P(x',t)(A(x') + B(x'))e^{ikx'} \\ & + 2i\int_0^\infty dz\sin(kz)\lambda(z)\int_{-\infty}^\infty dx' P(x',t)(A(x') - B(x'))e^{ikx'}\,.\end{aligned} \tag{S12}$$

We now introduce the cosine and sine transforms

$$\lambda_c(k) \equiv\ 2\int_0^\infty dz\cos(kz)\lambda(z)\,, \quad \text{and} \quad \lambda_s(k) \equiv\ 2\int_0^\infty dz\sin(kz)\lambda(z)\,. \tag{S13}$$

Using the convolution theorem for the second term in Eq.(S12) and recalling the relation $A(x)+B(x) = 1$, see Eq.(S8), we obtain the simple expression

$$P(k, t+\Delta t) = \lambda_c(k)P(k,t) + i\lambda_s(k)[P(k,t) * (A(k) - B(k))]\,, \tag{S14}$$

where $*$ denotes convolution in Fourier space, as derived previously in Ref.[6].

1.3 Fokker-Planck equation for the angle difference ψ

We now use this framework for non-local jumps to obtain an equation describing the temporal evolution of the probability distribution for the angle ψ. In general, changes in ψ after each elongation or branching event depend on the changes in the local angle $\varphi - \varphi'$ as well as on the changes in the angle to origin $\theta - \theta'$ values. However, after a small number of steps taken from the origin, the θ-dependent terms become negligible compared to changes in the local angle φ. The probability distribution for

the jumps sizes of the angle difference ψ can therefore be approximated by that of the local angle φ. In fact, as we will show later, see Supplementary Fig. 2(B) and (C), the mean-squared displacements for these two angles will attain the same linear form for sufficiently large times, indicating that the free diffusion of these two angular variables follow the same dynamics in this regime. As illustrated in Supplementary Fig. 2(A), the jumps in the angle values are described by uniform distributions with maximal jump sizes determined by $\delta\varphi_e \simeq \psi_e = \pi/10$ and $\delta\varphi_b \simeq \psi_b = \pi/2$ for elongation and branching events, respectively. Denoting the branching and elongation probabilities by p_b and p_e, respectively, and the change in the angle difference after each jump by $\bar{\psi} = \psi - \psi'$, we can express the jump size distribution as

$$\lambda(\bar{\psi}) = \frac{p_e}{2\psi_e}\left[H(\bar{\psi}) - H(\bar{\psi} - \psi_e)\right] + \frac{p_b}{2(\psi_b - \psi_e)}\left[H(\bar{\psi} - \psi_e) - H(\bar{\psi} - \psi_b)\right], \tag{S15}$$

where for clarity we only express the positive part of the symmetric jump distribution, *i.e.* for $\bar{\psi} \geq 0$. Fourier cosine and sine transforms of $\lambda(\bar{\psi})$ are then given by

$$\begin{aligned} \lambda_c(k) &= \frac{p_e}{k\psi_e}\sin(k\psi_e) + \frac{p_b}{k(\psi_b - \psi_e)}(\sin(k\psi_b) - \sin(k\psi_e)), \\ \lambda_s(k) &= -\frac{p_e}{k\psi_e}(\cos(k\psi_e) - 1) - \frac{p_b}{k(\psi_b - \psi_e)}(\cos(k\psi_b) - \cos(k\psi_e)). \end{aligned} \tag{S16}$$

Now we use the Taylor expansions up to $\mathcal{O}(k^3)$ for the cosine and sine functions to obtain:

$$\lambda_c(k) \simeq 1 - k^2 D, \quad \text{and} \quad \lambda_s(k) \simeq k\mu \tag{S17}$$

where

$$D \equiv \tfrac{1}{6}\left(p_b(\psi_b^2 + \psi_b\psi_e) + \psi_e^2\right), \quad \text{and} \quad \mu \equiv \tfrac{1}{2}\left(p_b\psi_b + \psi_e\right) \tag{S18}$$

can be defined as the diffusion and mobility/advection coefficients based on microscopic variables. Inserting these expressions into Eq.(S14) and taking the inverse Fourier transform leads to

$$P(\psi, t + \Delta t) - P(\psi, t) = D\partial_\psi^2 P(\psi, t) - \mu\partial_\psi[P(\psi, t)(A(\psi) - B(\psi))], \tag{S19}$$

We must now define A and B to model the external field which acts to reduce the alignment angle $|\psi|$ at each jump. Recalling our discussion on a radial external field, we introduce a sinusoidal force as described by Eq.(S5). Because $A(\psi) + B(\psi) = 1$, see Eq.(S8), and we want the drift on the particle to be determined by $A(\psi) - B(\psi) = -f_c\sin(\psi)$, we get

$$A(\psi) \equiv [1 - f_c\sin(\psi)]/2, \quad \text{and} \quad B(\psi) \equiv [1 + f_c\sin(\psi)]/2, \tag{S20}$$

Inserting these expressions into Eq.(S19), and assuming identical mean stepping times $\tau \equiv t/n$ in the limit of large step numbers n, we obtain the Fokker-Planck equation

$$\partial_t P(\psi, t) = D\partial_\psi^2 P(\psi, t) + \mu\partial_\psi[P(\psi, t) f_c \sin(\psi)] \tag{S21}$$

In the steady state we impose no-flux boundary conditions such that

$$\partial_\psi P^{\rm st}(\psi) = -\frac{\mu f_c}{D}\sin(\psi)P^{\rm st}(\psi). \tag{S22}$$

At steady state, using the ansatz $P^{\rm st}(\psi) = C\exp\left(\frac{\mu f_c}{D}\cos(\psi)\right)$ with constant C determined by the normalization condition $\int_{-\pi}^{\pi} P^{\rm st}(\psi)d\psi = 1$, as well as the symmetry of the cosine function, this predicts that $P^{\rm st}(\psi)$ should follow a von Mises distribution

$$P^{\rm st}(\psi) = \frac{1}{2\pi I_0(\nu)}\exp\left(\nu\cos(\psi)\right), \tag{S23}$$

where

$$I_0(\nu) = \pi^{-1} \int_0^{\pi} \exp(\nu \cos(\psi)) d\psi \tag{S24}$$

is the modified Bessel function of the first kind of order zero and

$$\nu \equiv \frac{\mu f_c}{D} \tag{S25}$$

takes the role of the concentration parameter of the von Mises distribution. This is a central result of our analytical model, which we confront to experimental data in the main text. Importantly, this predicts the distribution of angles up to a single rescaled parameter ν, which in analogy to the Péclet number quantifies the respective contribution of advection (arising from an extrinsic guiding field) to diffusion (arising from the intrinsic stochasticity of branching/elongation events) in the Fokker-Planck equation.

Note that the variance of the angle distribution ψ at steady state is thus directly related to this rescaled parameter:

$$\sigma^2 = 1 - \mathcal{R}(\nu), \quad \text{with } \mathcal{R}(\nu) \equiv \frac{I_1(\nu)}{I_0(\nu)}, \tag{S26}$$

whereas the circular standard deviation takes the form [7]

$$\sigma_{circ.} \equiv \sqrt{-2 \ln(\mathcal{R}(\nu))}, \tag{S27}$$

which deviates from the "linear" expression $\sqrt{1 - \mathcal{R}(\nu)}$. Note that, unlike the latter, Eq. (S27) diverges for $\nu \to 0$ and therefore does not provide a reliable metric for sufficiently spread out distributions. For the parameter regime we investigate here, however, it is an adequate estimator for the standard deviation of the underlying von Mises distribution. It is instructive to compare Eq. (S27) with the standard deviation of the equilibrium distribution for a particle in a harmonic potential, which corresponds to a normal distribution for ψ and depends on the external field f_c as

$$\sigma_{normal} \propto \sqrt{\frac{D}{\mu f_c}}. \tag{S28}$$

For sufficiently large values of ν, the von Mises SD given by Eq. (S27) exhibits a power-law behavior with a slightly larger decay coefficient, see Fig.1 (F) in the main text for a comparison of the two scaling laws.

2 Supplementary Note 2: Simulation details

Next, we briefly summarize the details for the numerical algorithm to simulate branching and annihilating random walks (BARWs) in the absence of external field or self-avoidance.

2.1 Branching and annihilating random walks (BARWs)

Similar to Ref.[8], we define active tips that will elongate and branch randomly, taking discrete steps of unit size $\ell = 1$ per discrete time interval $\tau = 1$ and leaving behing inactive branch segments that remain immobile at coordinate $\mathbf{r} \equiv (x, y)$ on a two-dimensional plane. When an active tip comes in close proximity of an inactive branch segment, *i.e.*, when the inactive branch is within an annihilation radius R_a of the active tip, the latter will become inactive and immobile. We choose a rather small annihilation radius of $R_a = 1.5\ell$ to mimic contact-dependent membrane recognition. Note that the specific choice of the annihilation radius in two dimensions does not influence the overall topology (up to global rescaling) of the networks because intersection of two branches occurs with probability 1 for all sufficiently small radii.

Simulations start with a single active tip at an initial position $\mathbf{r}_0 \equiv \mathbf{r}(\tau = 0) = (0, y_0)$ with a pre-defined polarity $\mathbf{p}_0 \equiv (1, 0)$, *i.e.*, which is directed horizontally towards the right (higher x values), and proceed until a certain maximal time $\tau_{\max} = 200\tau$ is reached. Because the active tips take a single step at each time interval τ, the maximal time $\tau_{\max}$ of the simulation also determines the maximal distance $R_{\max}$ from the origin $\mathbf{r}_0$ that the last surviving active tips can attain. For the experimental data, this parameter is therefore strongly constrained by the geometrical properties (overall size and shape) of the fin, which we determine by counting the *maximal* number of steps in the coarse-grained reconstruction of the filaments, see Supplementary Note 3.1. The initial polarity of the starting active tip allows us to define an initial *local angle* $\varphi_0 = 0$ with respect to the x-axis associated with the tip. The key parameter in this simulation setup is the branching probability p_b, which will determine the frequency of branching events and thus also the final density of the network.

The (i) elongation and (ii) branching events for the active tips are implemented in the simulation as follows: At each time point t, we draw n_a (pseudo-)random numbers $r_j \in (0, 1)$ with $j = 1, \ldots, n_a$, where n_a is the number of active tips at t. For each active tip, the elongation or branching occurs for $r_j > p_b$ or $r_j \leq p_b$, respectively. (i) When an active tip at coordinate $\mathbf{r}$ with local angle φ undergoes an elongation event, it takes one step "forward" such that its new local angle φ' will take a random value uniformly distributed between $\varphi \pm \delta\varphi_e$ with $\delta\varphi_e = \pi/10$. The latter rule leads to a small rotational diffusion of the tip during elongation events. The new position of the active tip will then be given by $\mathbf{r}' = \mathbf{r} + (\ell\cos(\varphi'), \ell\sin(\varphi'))$. Note that for $\delta\varphi_e = 0$ the rotational diffusion vanishes and the random walk becomes infinitely persistent. (ii) When an active tip with local angle φ undergoes a branching event, it will produce two new active tips at positions $\mathbf{r}'_1 = \mathbf{r} + (\ell\cos(\varphi'_1), \ell\sin(\varphi'_1))$ and $\mathbf{r}'_2 = \mathbf{r} + (\ell\cos(\varphi'_2), \ell\sin(\varphi'_2))$, respectively, and will become inactive itself. The local angles φ'_1 and φ'_2 of the two new active tips respectively take values uniformly distributed in $[\varphi + \delta\varphi_e, \varphi + \delta\varphi_b]$ and $[\varphi - \delta\varphi_e, \varphi - \delta\varphi_b]$ with $\delta\varphi_b = \pi/2$, *i.e.*, the two new tips will be located on the two different sides of the line determined by the polarity vector $\mathbf{p} = (\ell\cos(\varphi), \ell\sin(\varphi))$ of their parent while preserving a minimal angle of $\Delta\varphi'_{min} = \varphi'_1 - \varphi'_2 = 2\delta\varphi_e = \pi/5$ between each other. The latter rule enforces a minimal distance between the two new tips to reduce the frequency of immediate annihilation of the two new tips. Furthermore, we also found that 85% of bifurcation angles obtained from the experiments were also distributed within the range $[\pi/5, \pi]$, which justified this choice of the simulation setup. Supplementary Fig. 2(A) illustrates the elementary steps implemented in the simulation for the branching, elongation and annihilation of the active tips.

To test our analytical predictions for the "free" diffusion of branch segments, we first ran simulations for single branches that perform elongation and branching jumps without generating additional new active tips. We found that the mean-squared displacements of the final local angle φ of single branches at time $t_{\max}$ closely follow the relation $\langle \Delta\varphi(t_{\max})^2 \rangle = 2Dt_{\max}$ with the diffusion constant D given by the "microscopic" expression Eq. (S18), see Supplementary Fig. 2(B). We also obtained the same relation for the mean-squared displacements of the *angle differences*, *i.e.*, $\langle \Delta\psi(t_{\max})^2 \rangle = 2Dt_{\max}$ for large values of $t_{\max}$, see Supplementary Fig. 2(C). The latter results justified our hypothesis that for sufficiently large times, the instantaneous jumps in the angle difference values ψ are dominated by the changes in the local angle φ, and thus do not depend on the angle θ to origin.

For the full BARW simulations, we could then set a branching probability p_b, the initial position $\mathbf{r}_0$, and the initial polarity vector $\mathbf{p}_0$, and produced branching networks that radially grow in all directions over time with active tips forming a front, leaving inactive branches of constant density in the "inner" regions. When the network growth is constrained within a spatial region delineated by fixed boundaries, we observe an apparent directionality as predicted previously [8], see Supplementary Fig. 3(A). However, tissue growth in the absence of spatial boundaries always remains isotropic, see Supplementary Fig. 3(B), which also remains valid for branching networks with strong self-avoidance. We concluded that one needs to define an external field to guide the tips for a directed (anisotropic)

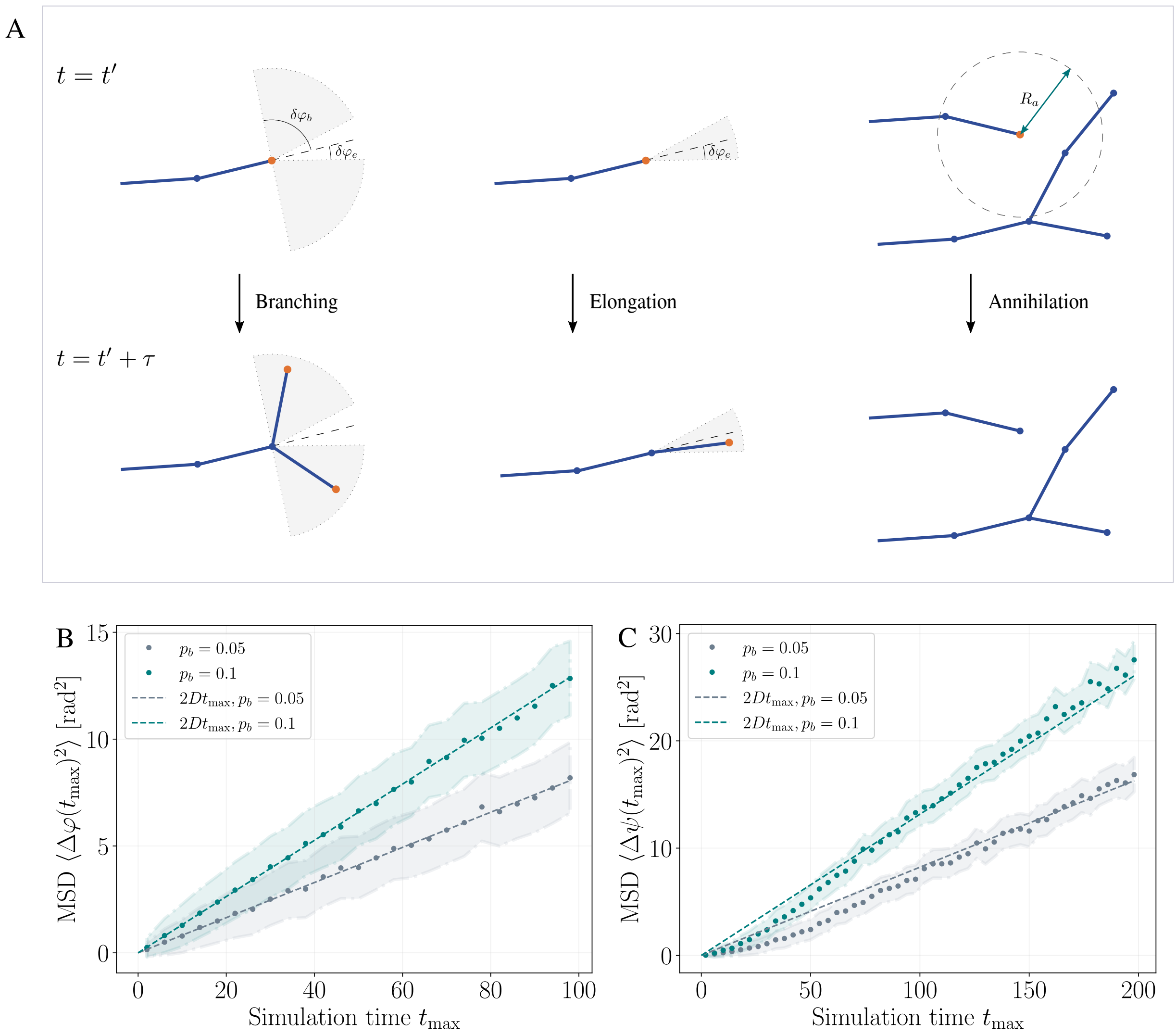

Supplementary Figure 2: **Schematics and quantifications of the two mechanisms leading to angular diffusion of branches.** (A) Elementary steps in the implementation of the BARW simulations: (Left) With a given *a priori* branching probability p_b, an active tip (orange node) of a branch segment at time t' generates two additional active tips at the next time step $t' + \tau$, where τ is a discrete time interval (set to $\tau = 1$ in the simulations). The angles between the new branch segments with the previous segment take values uniformly distributed in $\pm[\delta\varphi_e, \delta\varphi_b]$ (gray shaded areas), where we use $\delta\varphi_e = \pi/10$ and $\delta\varphi_b = \pi/2$. (Middle) Elongation of an existing branch segment occurs with the probability $p_e = 1 - p_b$ and leads to a small rotational diffusion of the branch confined within a cone determined by the angle $2\delta\varphi_e$ (gray shaded area). (Right) If an active tip comes in close proximity of existing inactive branch segment (blue nodes) it will annihilate, *i.e.*, become inactive. The local neighborhood (dashed circle) for sensing inactive branches is determined by an annihilation radius R_a (green arrow). (B) Simulated trajectories of a single branch segment freely diffusing without external potential and annihilation displays a mean-squared displacement (MSD) for the local angle φ that obeys the relation $\langle \Delta\varphi(t_{\max})^2 \rangle = 2Dt_{\max}$ with the diffusion constant D predicted by the microscopic theory, see Eq. (S18). (C) MSD for the angle difference ψ of a single branch segment approaches the analytical prediction for large times ($t_{\max} \geq 100$). Plot markers represent the mean values and the shaded regions denote circular SDs around the mean values ($n = 1000$ and $n = 2000$ runs for (B) and (C), respectively).

spatial invasion such as seen in the experiments.

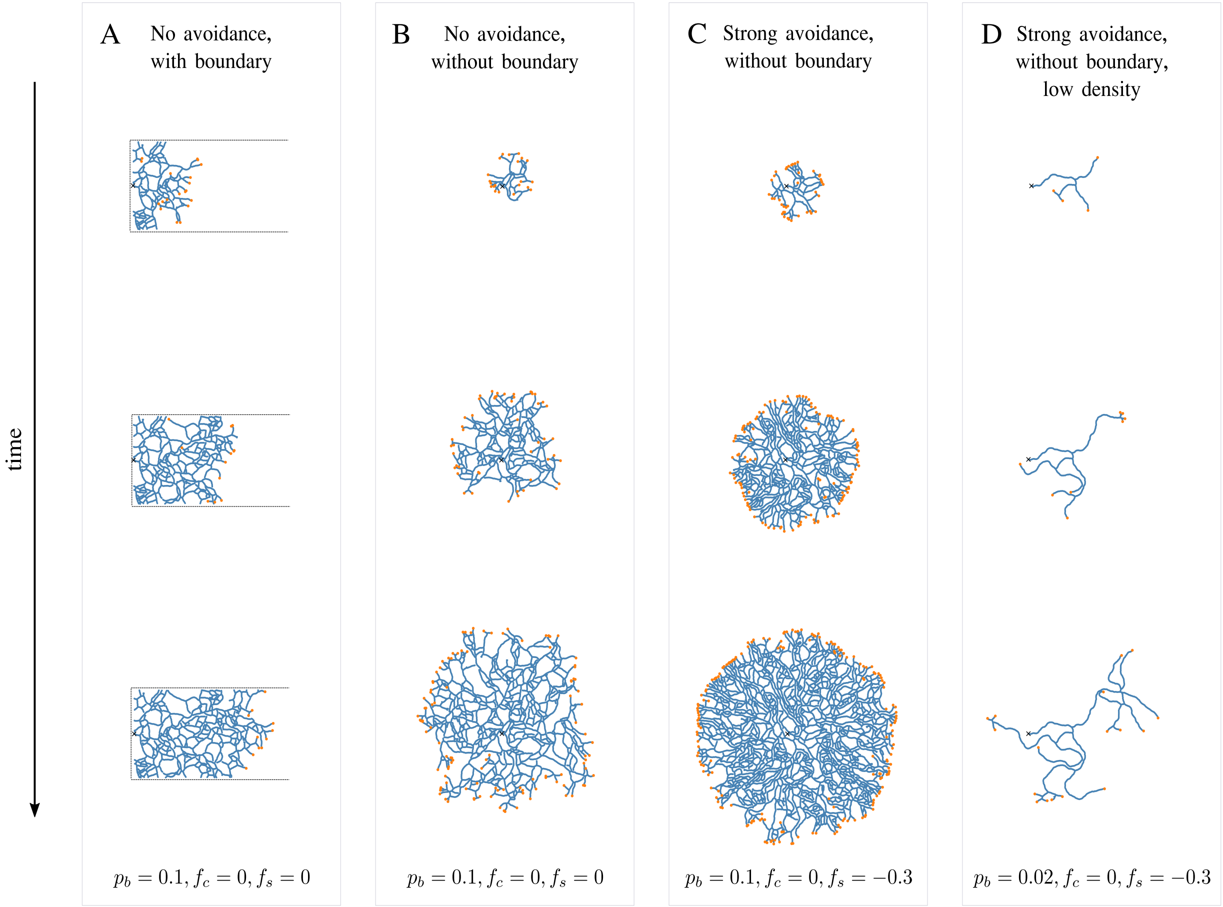

Supplementary Figure 3: **Time evolution of BARWs in the absence of an external field**. All networks start growing from the initial point denoted by the cross symbol (black) for $f_c = 0$. (A) Simulation of BARWs confined in a bounded region (represented by the dashed rectangular half-open box) exhibits directed growth of the branched network effectively towards the opening on the right. (B) When the boundary is removed, the branching network grows isotropically in all directions, where active tips leave passive branch segments in the inner regions of the network. (C) BARWs with a rather strong self-avoidance ($f_s = -0.3$) generate a dense network with efficient space-filling (as described in Fig. 5 of the main text), where active tips seem to define a sharp front that propagates isotropically. (D) Reducing the branching probability results in a network with a small number of active tips and a well-defined front does not emerge. Despite the strong self-avoidance, an anisotropic growth cannot be observed in this purely local, self-organized model in the absence of external field.

2.2 BARWs with external guidance and/or self-avoidance

In this subsection, we describe different possible microscopic ways to implement external guidance in the simulations of branching and annihilating random walks, and show importantly that the two give rise to the same qualitative predictions in the continuum model in terms of overall branch alignment.

2.2.1 Microscopic mechanisms for implementing external guidance

Observations of the movement of single neuronal tips in the presence of chemical cues (such as Netrin) indicate that the neuronal tips can reorient themselves even without neighboring tips or other branches in close proximity [9]. This change in the directionality can be effectively modelled as a displacement of the tip towards the external field, as we discussed in Supplementary Note 1.1.1 and represent in Supplementary Fig. 1. Note that, however, such a displacement would lead to an instantaneous shift (jump) in the angle difference value ψ of the active tip due to the external field in contrast with the the

continuum model developed in Supplementary Note 1.3, according to which the external field influences the *jump probabilities* for the angle difference ψ *via* the forward and backward bias terms defined in Eq. (S20). We will therefore discuss these two different ways of implementing the external field, and show that they generically result in the same behavior and the angular alignments follow the same statistics up to a constant prefactor for the field strength.

External field *via* biased probabilities. To closely follow the continuum model, we first implemented biased jump probabilities in the simulation as follows: For each elongation event, we weighted the elongation probability $p_e = 1 - p_b$ by the bias terms such that a "forward" and "backward" step that respectively increases or decreases the angle difference value ψ occurs with the probability $p_e\,A(\psi)$ and $p_e\,B(\psi)$ with $A(\psi) = [1 - f_c \sin(\psi)]/2$ and $B(\psi) = [1 + f_c \sin(\psi)]/2$ as previously defined in Eq. (S20). Here, the dimensionless parameter f_c denotes the strength of the external field. Because $A(\psi)+B(\psi) = 1$, the joint probability of a forward and backward elongation event is equal to p_e which is the *a priori* value fixed by setting the branching probability p_b. For instance, for an active tip with a positive angle difference value $0 < \psi < \pi$, this implementation of the biased jumps implies that it is more likely for the tip to reduce its local angle than to increase it upon elongation. For branching events we do not implement the effect of the external field f_c on the angle values of the tip because the bifurcation event in the simulation corresponds to two opposite jumps in the local angle values (the two tips branch into different sides of the polarity vector). In contrast, the jumps corresponding both to elongation and branching events in the continuum model are influenced by the external field *via* the bias terms $A(\psi)$ and $B(\psi)$. Even though we omit the bias for the branching events, we obtained a good agreement with the analytical predictions for the angle distributions obtained using this simulation setup, as shown in the main text.

External field *via* tip displacement. We also wanted to explore the modelling of the external field in a different way, to show the generality of our approach and the insensitivity to the details of the microscopic tip behavior, and thus implemented guidance *via* tip displacements. In this simulation setup, for a radial external field, the active tips would now be displaced at each time step by a factor $f_c\,\mathbf{p}_c$, where $\mathbf{p}_c \equiv (\ell\cos(\theta), \ell\sin(\theta))$ is the unit vector pointing along the field lines, and θ denotes the angle to origin of the tip before displacement. To explore an axial field, e.g. oriented along the positive x axis, one can define the unit vector for the field polarity to be $\mathbf{p}_c^x \equiv (\ell, 0)$. In this implementation of the external guidance, the field thus directly "corrected" the directionality of the tip migration, as opposed to the "effective correction" which arose from the biased transition probabilities of the previous approach. Note that, the branch segments undergo a small rotational diffusion upon elongation with a noise determined by the elongation angle $\delta\varphi_e$, as illustrated in Supplementary Fig. 2A. The displacement by the external field is performed *subsequently* on the tips such that branch orientations are not completely deterministic. Even though these two ways of implementing the external field are quite different, we found that they generated both qualitatively and quantitatively very similar network structures, see Supplementary Note 2.5 for a comparison of the results. Crucially, we observed that one only needs to tune the external field strength f_c by a constant prefactor α to obtain similar results using the two different algorithms.

2.2.2 Implementing self-avoidance

An additional feature observed in some branching tissues is the presence of self-avoidance of growing tips, *e.g.*, as clearly evidenced in morphogenesis of starburst amacrine cells [10], where active tips sense, and are repulsed by, existing branch segments in close proximity. For neuronal branching it was shown that this self-avoidance of the growing dendrites is based on isoform recognition of the membrane

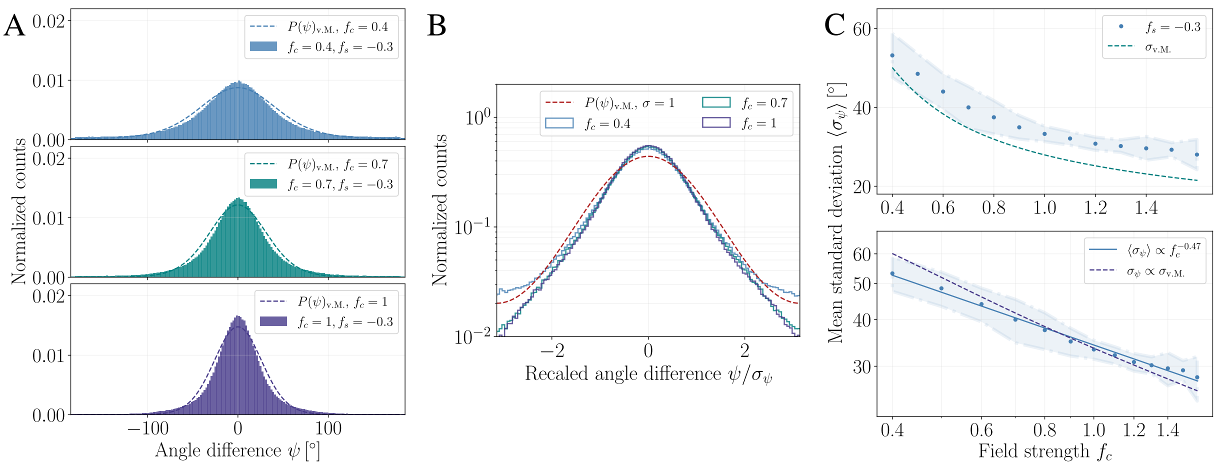

Supplementary Figure 4: **Effects of strong self-avoidance ($f_s = -0.3$) on the angular alignment for BARWs in a radial external cue.** (A) Normalized histograms for the angle difference ψ for different values of the external field strength f_c. Dashed lines represent the theoretical predictions given by the von Mises distribution with the concentration parameter $\nu = \frac{\mu f_c}{D}$. Presence of strong self-avoidance leads to narrower distributions for ψ and to a strong deviation from the analytical prediction for large f_c, in contrast with the case without self-avoidance, see Fig. 3b in the main text. (B) Normalized histograms for ψ rescaled by their corresponding mean standard deviations (SDs) σ_ψ (solid lines) compared to the von Mises distribution with unit SD (dased line) exhibit a sharper peak and pronounced deviations at the tails for large f_c. (C) Mean SDs of the normalized histograms for ψ decay monotonically as a function of f_c and attain larger values than the SDs corresponding to the von Mises distribution (dashed line, top). (Bottom) Scaling of the SDs obeys a power-law $\langle\sigma_\psi\rangle \propto f_c^{-0.47}$ close to the scaling precited by the von Mises distribution (dashed line). Averages were taken over $n = 100$ simulation runs for each parameter choice.

proteins [11]. We modelled this effect by considering that active tips sense an average density vector $\mathbf{p}_s$ [8] depending on the number of branch segments within a certain radius R_s of "self-recognition":

$$\mathbf{p}_s \equiv \frac{\Sigma_j(\mathbf{r}(t) - \mathbf{r}_j(t))}{|\Sigma_j(\mathbf{r}(t) - \mathbf{r}_j(t))|}, \quad \text{with} \quad |\mathbf{r}(t) - \mathbf{r}_j(t)| < R_s\,. \tag{S29}$$

Here we denote the position of the active tip at time t by $\mathbf{r}(t)$ and that of the remaining particles within R_s by $\mathbf{r}_j(t)$. Branch segments ("particles") that belong to the same branch do not contribute to the sum, i.e. the active tip does not "sense" inactive segments from its own branch. The density vector $\mathbf{p}_s$ will thus define a normalized vector pointing *away* from nearby particles. We can now define a self-recognition force by $-f_s\mathbf{p}_s$, where the dimensionless parameter f_s determines the strength of interaction with $f_s > 0$ and $f_s < 0$ corresponding to attraction and repulsion, respectively. Note that this self-repulsion force can effectively correspond to sequential recognition vs. retraction events such as seen in neuronal dendrites and as modelled *e.g.* in Ref.[12]. In contrast with the latter study, here we do not focus on the statistics of the retraction events and could therefore describe the self-recognition *via* a more simplified effective modelling. The position of the active tip after displacement by this self-recognition force will then simply change to $\mathbf{r}'(t) = \mathbf{r}(t) - f_s\mathbf{p}_s$. However, in order to preserve the step length ℓ, we will also correct this position such that the distance between the final position $\mathbf{r}^*(t)$ after displacement and the position *at the previous time step* is equal to ℓ, *i.e.*, $|\mathbf{r}^*(t) - \mathbf{r}(t-\tau)| = \ell$. Note that, the new orientation of the branch segment does not align with $\mathbf{p}_s$ but with the vector $\mathbf{r}^*(t) - \mathbf{r}(t-\tau)$. Supplementary Fig.5(A) illustrates the quantitites and displacement rule used in the implementation of self-avoidance.

2.3 Estimating the average opening angle

As briefly discussed in the main text, the area of invasion of a branching network in a radial external field will be proportional to the average opening angle $\bar{\theta}$ between the outermost branch segments of the network. To quantify this, we conjectured that the overall opening angle is very likely to be dependent on the local dynamics *at the growing boundary* of a branching network. Indeed, using simple geometric arguments for the changes in the position and angles of the active tip (sine rule for the triangles defined by the origin and tip positions before and after tip displacement), similar to our analysis in Supplementary Note 1.1.1, we infer

$$\frac{\sin(\delta\theta)}{\ell} = \frac{\sin(\gamma)}{r^*(t-\tau)\ell} \tag{S30}$$

and

$$\frac{\sin(\Delta\varphi)}{f_c\,\ell} = \frac{\sin(\gamma)r^*(t)}{(r(t)+f_c)\ell}\,, \tag{S31}$$

where $\Delta\varphi \equiv \varphi - \varphi^*$ is the change in the local angle after the displacement by the external field f_c at time t, $r(t)$ and $r^*(t)$ denote respectively the radial distance of the active tip before and after the displacement by the external field, and $\delta\theta \equiv \theta^*(t) - \theta(t-\tau)$ is the change in the angle to origin values between two consecutive time intervals, see Supplementary Fig.5(B) for an illustration. For tip positions sufficiently far from the origin, we can approximate for all distances $r = r^*(t) = r^*(t-\tau) = r(t)$, and after rearranging the above equations we obtain:

$$\delta\theta \simeq \frac{f_c + r}{f_c\,r^2}\sin(\Delta\varphi)\,, \tag{S32}$$

For the dynamics "orthogonal" to the growth direction that we are interested here, the changes in the radial distance between consecutive time steps can be neglected and thus described effectively by a single parameter r replacing the temporal parameter t. After these simplifying assumptions (and assuming $\delta\theta/dr$ is a well-defined infinitesimal), we can integrate Eq. (S32) to obtain

$$\bar{\theta}(r) = 2\int dr\delta\theta \simeq 2\sin(\Delta\varphi)\left(\frac{\log(r)}{f_c} - \frac{1}{r}\right)\,, \tag{S33}$$

where we used the symmetry for the two growing boundaries of the branched network (prefactor 2). Arguing that $\Delta\varphi$ is likely to be dominated by a small constant for large times, we can approximate the prefactor $\sin(\Delta\varphi) \simeq \chi$ with a free parameter χ. For large times, the second term in Eq. (S33) inversely proportional to r becomes smaller and thus we arrive at Eq. (3) of the main text.

2.4 Simulation results for BARWs with self-avoidance

2.4.1 Strong self-avoidance without external field

To clarify whether directed growth can arise in a self-organized way in the absence of an external field just by the self-avoidance mechanism, we performed simulations with a strong self-avoidance of $f_s = -0.3$ but setting the external field strength to $f_c = 0$. In the density regime set by a branching probability of $p_b = 0.1$, we again observed isotropic growth of the networks, see Supplementary Fig. 3(C). However, due to the the rather strong self-avoidance potential, the final network exhibited a qualitatively much "ordered" structure, in agreement with the predictions in [8]. Interestingly, the active tips formed a sharper propagating front in contrast to the case without self-avoidance. We finally analyzed the low-density case by reducing the branching probability to $p_b = 0.02$ to see if the network could preserve its pre-defined directionality uninterrupted by the "outwards push" due to branching events. However, such a self-organized anisotropic growth also could not be obtained for the low-density case, see Supplementary Fig. 3(D), which indicated that an external field is required to break the isotropy in the tissue growth.

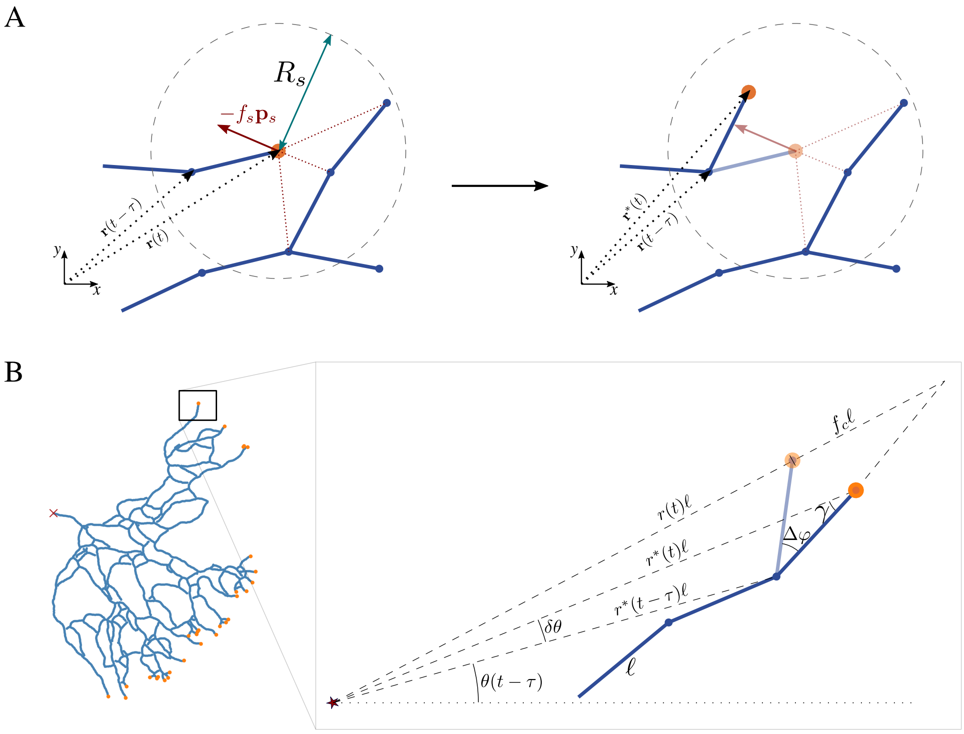

Supplementary Figure 5: **Schematics for the self-avoidance rule and estimation of the opening angle.** (A) The active tip (orange node) with position vector $\mathbf{r}(t)$ (dotted arrow) at time t senses its neighboring branch segments within a radius R_s (dashed circular line). Its position at the previous time step $t-\tau$ is denoted by $\mathbf{r}(t-\tau)$. The distances between the active tip and its neighbors (red dotted lines) define a unit self-interaction vector $\mathbf{p}_s$, see Eq.(S29), that is rescaled by the self-avoidance parameter f_s (red arrow). (Right panel) After the displacement due to the local density of neighboring branches, the final position of the active tip will be determined by the vector $\mathbf{r}^*(t)$ which preserves the length of the branch segment by $|\mathbf{r}^*(t) - \mathbf{r}(t-\tau)| = \ell$. (B) To estimate the opening angle $\bar{\theta}$ of a branched network (left), we focus on the active tip at the boundary of the network (orange tip in the boxed region): Tip displacement by the external field with strength f_c changes the local angle φ of the tip by a factor $\Delta\varphi$ and its distance to origin changes from $r(t)\ell$ to $r^*(t)\ell$ (dashed lines to the tip). The angle to origin θ at time t after displacement is then given by $\theta^*(t) = \theta(t-\tau) + \delta\theta$, where $\theta(t-\tau)$ denotes the angle to origin at the previos time step $t-\tau$. For a tip sufficiently distant from the origin, we can approximate $r = r(t) = r^*(t) = r^*(t-\tau)$. Using the sine rule for the triangles defined by the origin and the tip positions before and after displacement, we obtain the relations leading to Eq.(S32).

2.4.2 Strong self-avoidance with external field

Here we provide further results for simulations with a high self-avoidance potential and in the presence of external guidance. For increasing values of f_s, we observed that the density and the space-filling properties of the network markedly increased, as shown in Fig. 5 in the main text. We also qualitatively observed that the networks with high self-avoidance exhibit better alignment with the external field. This increased alignment is indeed reflected in the angle distributions, where the distributions for the angle difference become narrower compared with those from simulations without self-avoidance, see Supplementary Fig. 4. The mechanism can presumably be linked to the high density of the network and its space-filling properties, where self-avoidance competes against annihilation events, thus the network effectively generates more segments in dense regions that are forced to be aligned with the external field. Supporting this hypothesis further, we see faster decaying tails for large values of f_c and for strong-repulsion, as exhibited by the rescaled angle difference histograms in Supplementary Fig. 4(B).

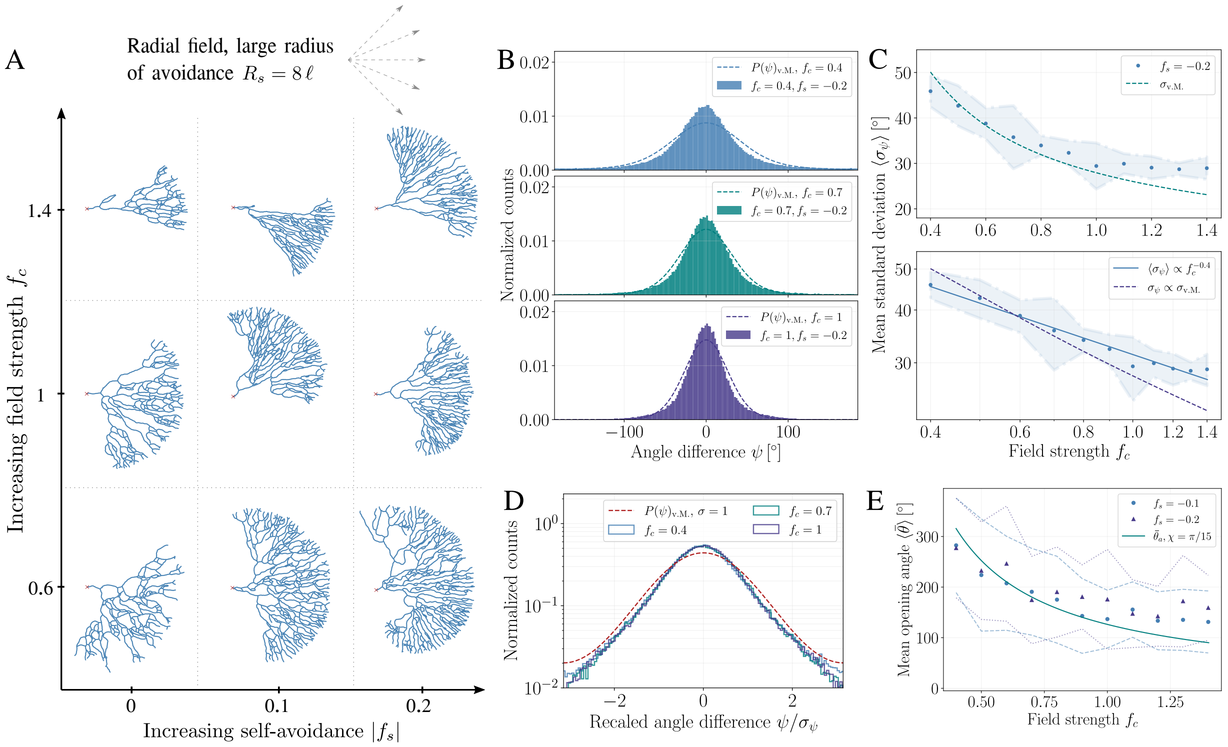

Supplementary Figure 6: **Effects of a large radius of self-repulsion ($R_s = 8\ell$) on the alignment and morphology of branching networks.** (A) Morphology diagram of branching networks in a radial field. For strong self-repulsion, individual branches exhibit pronounced alignment with characteristic gaps larger than those seen for smaller radius of avoidance as used in the main text (see Fig. 2a in the main text with $R_s = 3\ell$). (B) Normalized histograms for the angle difference ψ for $f_s = -0.2$ display sharper peaks than those of the von Mises distributions predicted by theory (dashed lines). (C) Mean standard deviations (SDs) for ψ are close to theoretically predicted values (dashed line, top) but their scaling with increasing field strength f_c deviates rather strongly from the scaling relation for the SD $\sigma_{\mathrm{v.M.}}$ of the von Mises distribution (bottom). (D) Histograms for ψ rescaled by their SDs (solid lines) illustrate sharper peaks and faster decaying tails compared to the von Mises distribution with unit SD (dashed line). (E) Mean opening angles $\langle\bar{\theta}\rangle$ for branching networks with a large radius of repulsion for a repulsion strength of $f_s = -0.1$ (circular markers) and $f_s = -0.2$ (triangular markers) as a function of the field strength f_c. For large f_c, the opening angles saturate and deviate from the predicted decay $\bar{\theta}_a$ (solid line), in contrast with the case for a small radius of avoidance (compare Fig. 4 in the main text). For every f_c, averages were taken over $n = 20$ and $n = 50$ simulation runs for the cases $f_s = -0.2$ and $f_s = -0.1$, respectively.

2.4.3 Large radius of self-avoidance

To explore the alternative of self-interactions that are controlled at larger distances (for instance e.g. longer retraction events after contact-mediated self-recognition) we set a large radius of self-recognition $R_s = 8\ell$ as compared with the radius $R_s = 3\ell$ we used otherwise. Interestingly, even on a visual level, we could observe strongly aligned branches in the morphology diagram, see Supplementary Fig. 6(A), which became quite pronounced for a large value of self-repulsion ($f_s = -0.2$, rightmost column). Analysis of the angle distributions revealed that this case indeed leads to large deviations from the analytical predictions, and the standard deviations decay also rather slowly with increasing f_c, see Supplementary Fig. 6(B) and (C). For large f_c, these effects of self-repulsion become rather dominant and lead to a saturation of the mean opening angles $\langle\bar{\theta}\rangle$ of the networks as shown in Supplementary Fig. 6(E). Together, these results indicate that such long-range self-interaction effects can indeed influence the angular alignment and morphology of the final networks, especially in the presence of strong external potentials (although our data indicates that this it not the experimentally relevant limit, see

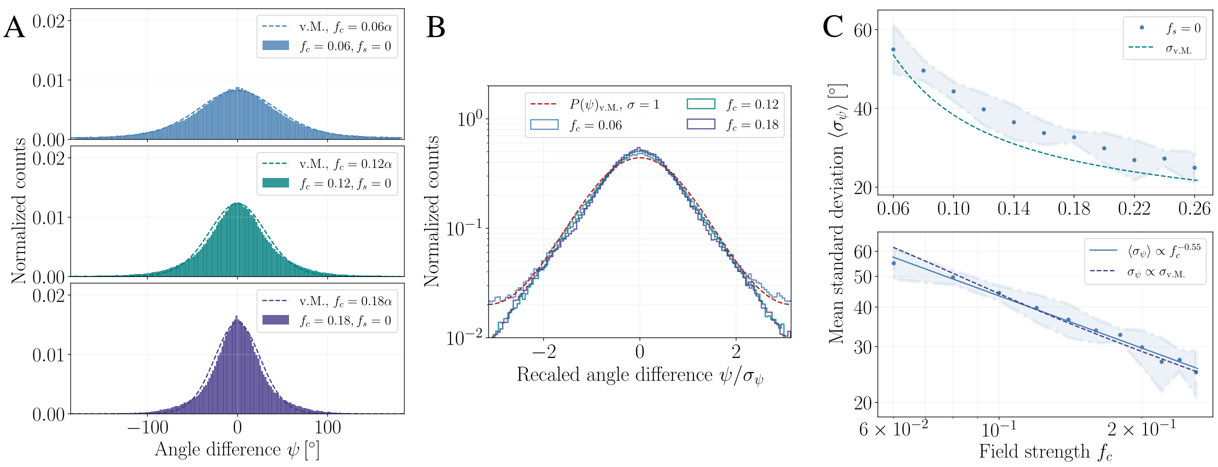

Supplementary Figure 7: **Statistics of BARWs with external guidance *via* deterministic displacement of active tips.** (A) Normalized histograms for the angle difference ψ for different values of the external field strength f_c are well-approximated by von Mises distributions (dashed lines) with a concentration parameter $\nu = \frac{\mu}{D}\alpha f_c$ with an additional prefactor $\alpha = 6$ used as a fit parameter. (B) Histograms for ψ rescaled by their corresponding standard deviations (SDs) are well-described by the von Mises distribution with unit SD (dashed line). For large f_c the tails of rescaled histograms (solid lines) decay faster than that of the von Mises distribution. (C) Mean SDs $\langle\sigma_\psi\rangle$ of the angle difference distributions decay monotonically with f_c (top) with values close to the analytical prediction (dashed line), and are consistent with a power-law relation $\langle\sigma_\psi\rangle \propto f_c^{-0.55}$ (bottom) well-approximated by the analytical relation $\sigma_{\text{v.M.}}$ corresponding to the von Mises distribution (dashed line). Averages were taken over $n = 100$ simulation runs for each parameter choice.

Fig. 5 of the main text, this would be an interesting metric to test in other branched organs).

2.5 Simulation of BARWs with external guidance *via* tip displacement

Next, we performed simulations with the alternative implementation of the external guidance *via* direct tip displacements, as we briefly described in Supplementary Note 2.2. First, we qualitatively observed a high similarity between the final network topologies generated using the two methods for different values of the external field strength. We then quantified this similarity by analyzing the angle distributions and the scaling of the alignment fluctuations, see Supplementary Fig. 7. We found that the two methods generically provide the same statistics after tuning the field strength f_c by a constant prefactor α. Note that for different choices of the branching probability, this pre-factor needed to be changed slightly but recovered the statistics for different f_c values. To probe the relevance of direct tip displacement-based simulations, live-imaging dataset would be needed to investigate more systematically the microscopic mechanisms of tip elongation, repulsion and guidance.

2.6 Simulation of BARWs with external guidance in three-dimensions

2.6.1 Generalization of the simulation setup

To probe the validity and generality of our main results, here we extend our simulation setup for branched structures growing in a three-dimensional (3D) geometry. To implement this, we switch to a co-moving spherical coordinate system with an origin located at the root of a given active branch segment, see Supplementary Fig. 8. The azimuthal Φ and polar Θ angles then uniquely determine the local orientation of the branch segment. To determine the rules for branching/elongation it is instructive to imagine a sphere of radius ℓ with its center at the *tip* of the active branch segment. On this sphere, we can now define a set of circles with centers lying on the local orientation vector

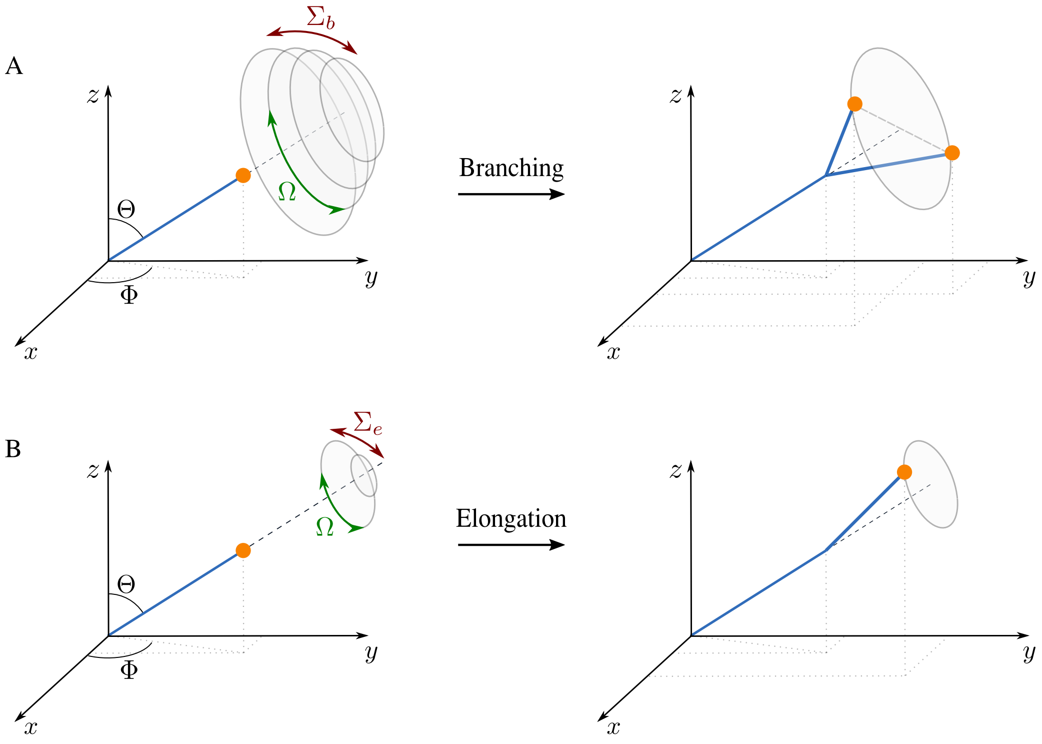

Supplementary Figure 8: **Schematic for the implementation of branching and elongation rules in a three-dimensional (3D) geometry**. In the local coordinate system of a branch segment with origin centered at the root of the segment, its local orientation is determined by the pair of polar Θ and azimuthal Φ angles. (A) For a tip (orange node) undergoing a branching event, one can parametrize a set of circles (gray lines) that lie on a sphere (with radius ℓ) around the tip and have centers located at the intersection with the orientation vector (dashed line) of the branch segment. The parameter Σ_b then sets the radius of such a circle and Ω can be used to determine the angular position on a chosen circle. After branching, the coordinates of one of the new tips will then be determined by the chosen set of Σ_b' and $\Omega' = \Omega_1$, whereas the position of the second tip will be determined by Σ_b' and $\Omega_2 = \Omega_1 + \pi$, i.e. it will be placed *opposite* the first one on the chosen circle. (B) Likewise, for a tip undergoing an elongation, its new position will be determined by the pair Σ_e and Ω', while Σ_e takes smaller values compared to Σ_b (only allowing circles with small radii) to enforce a small rotational diffusion of the tip during elongation.

of the branch segment, see gray circles shown in Supplementary Fig. 8 (left column). Starting from a parametrization of a circle around the "north pole" (around the z-axis) we can apply two rotations on it to obtain a general expression for Cartesian coordinates of all possible circles along a given local branch orientation vector (the latter fixed by the pair (Φ, Θ)) as a function of two new variables Σ and Ω as follows:

$$\begin{aligned} x &= \ell\left(\cos(\Omega)\left[\sin(\Sigma)\cos(\Theta)\cos(\Phi)\right] - \sin(\Omega)\left[\sin(\Sigma)\sin(\Phi)\right] + \cos(\Sigma)\sin(\Theta)\cos(\Phi)\right) , \\ y &= \ell\left(\cos(\Omega)\left[\sin(\Sigma)\cos(\Theta)\sin(\Phi)\right] + \sin(\Omega)\left[\sin(\Sigma)\cos(\Phi)\right] + \cos(\Sigma)\sin(\Theta)\sin(\Phi)\right) , \\ z &= \ell\left(\cos(\Sigma)\cos(\Theta) - \cos(\Omega)\sin(\Sigma)\sin(\Theta)\right) . \end{aligned} \tag{S34}$$

The two parameters Σ and Ω will then determine the new position of the tip after a branching or elongation event: (i) For a tip undergoing an elongation, we first choose a random value for $\Sigma = \Sigma_e$ uniformly distributed in $[0, \pi/10]$. This thus fixes the radius for the circle of the sphere where the new tip position will be located. We then pick a random value for Ω uniformly distributed in $[0, 2\pi]$ to determine the position of the new tip on the chosen circle. Note that, for $\Sigma = 0$ the tip elongates while preserving its previous orientation. (ii) For branching, we now pick a random value for $\Sigma = \Sigma_b$ uniformly distributed in $[\pi/5, \pi/2]$ because we find that circles with an opening angle Σ smaller than $\pi/5$ lead to frequent annihilation events of newly born progenies upon branching. After fixing Σ_b we

again choose $\Omega = \Omega_1$ uniformly distributed in $[0, 2\pi]$ to determine the position of one of the new tips on the chosen circle. The second tip is then placed *opposite* the first one by fixing an angle parameter $\Omega = \Omega_2 = \Omega_1 + \pi$, see Supplementary Fig. 8 for an illustration of these rules.

In the absence of external guidance, these rules then lead to isotropically growing branched networks in 3D, providing a similar phenomenology as seen in 2D. To explore the effects of external guidance, we now implement additional tip displacements along the field polarity at every time step, in analogy to our implementation described in Supplementary Note 2.2.1 for a 2D geometry. For the simplest case of axial (linear) guidance, e.g. for a field oriented towards the positive x-axis, we can define additional tip displacements determined by a factor $f_c \mathbf{p}_c^x$ where $\mathbf{p}_c^x \equiv (\ell, 0, 0)$ is the unit polarity vector of the external field. After the displacement we update the branch position such that its length with respect to its previous position is conserved as ℓ, and transform back to spherical coordinates (Φ, Θ) in the *local frame of reference of the active branch* to obtain the local orientation of the branch segment after tip displacement. Finally, self-avoidance can be implemented in complete analogy to 2D, with the only modification being that the tip can sense neighboring branch segments within a *sphere* of radius R_s around it, which will then lead to an additional displacement of the tip as described in Supplementary Note 2.2.2.

2.6.2 Results for BARWs with external guidance in a 3D geometry

We first wanted to see if our main results for BARWs with external guidance could be reproduced qualitatively in a 3D setting. Indeed, building a morphology diagram for different values of external guidance strength f_c (axial field along x) and self-avoidance f_s led to a qualitatively similar picture to the ones shown in Figs.1 & 2 in the main text: Increasing f_c systematically decreased the overall territory of the branched networks, whereas large self-avoidance seemed to only increase the local density of branch segments, minimally influencing the territory size, see Supplementary Fig. 9(A).

Next, to quantify these features, we generalized the opening angle $\bar{\theta}$ which we used as a proxy for territory size in 2D by defining a right circular cone with its apex positioned at the origin of the branched network. The altitude vector of this cone (vector connecting the apex to the center of its base) is chosen to be aligned with the average polarity of the branched network $\mathbf{p}_r \equiv \sum_j \mathbf{r}_j / |\sum_j \mathbf{r}_j|$, where $\mathbf{r}_j$ denotes the position vector of the j-th branch segment and j runs through all branch segments of the network. Using this construction, we could then define an opening angle $\bar{\theta}_{3D}$ of the cone that contains the entire branched network, where we determine $\bar{\theta}_{3D} \equiv max(\arccos(\mathbf{p}_r \cdot \mathbf{r}_j))$ after evaluating the dot product for each branch segment j. These estimated opening angles $\langle \bar{\theta}_{3D} \rangle$ (averaged over $n = 30$ simulations for each parameter choice) as a function of the external field strength f_c for vanishing ($f_s = 0$) and strong ($f_s = -0.3$) self-avoidance showed that, in complete analogy to 2D, self-avoidance indeed has a minimal influence on the overall territory of the branched networks in 3D, see Supplementary Fig. 9(B).

To test the generalization of our analytical predictions on the branch orientations and scaling, we first looked at the distributions of the azimuthal Φ and polar Θ angles of the branch segments for different choices of external field strength f_c and for vanishing self-avoidance ($f_s = 0$). Both angles provide measures of alignment with the polarity of the external field (here along the positive x-axis), where $\Phi = 0$ and $\Theta = \pi/2$ would correspond to a completely aligned branch segment. To compare these angles with our analytical predictions, we shifted these angles to take values around 0° (i.e. $\tilde{\Theta} \in [-90°, 90°]$ and $\tilde{\Phi} \in [-180°, 180°]$). Similar to our results from tip displacement-based simulations in 2D, see Supplementary Fig.7, the distributions for $\tilde{\Phi}$ and $\tilde{\Theta}$ could be well-approximated by von Mises distributions with a concentration parameter $\nu = \frac{\mu}{D} \zeta f_c$, where the prefactors $\zeta = \beta = 11$ (for $\tilde{\Theta}$) and $\zeta = \gamma = 8$ (for $\tilde{\Phi}$) were used as fit parameters, see Supplementary Fig. 9(C). Interestingly, the distributions remained markedly unchanged for strong self-avoidance $f_s = -0.3$, see Supplementary

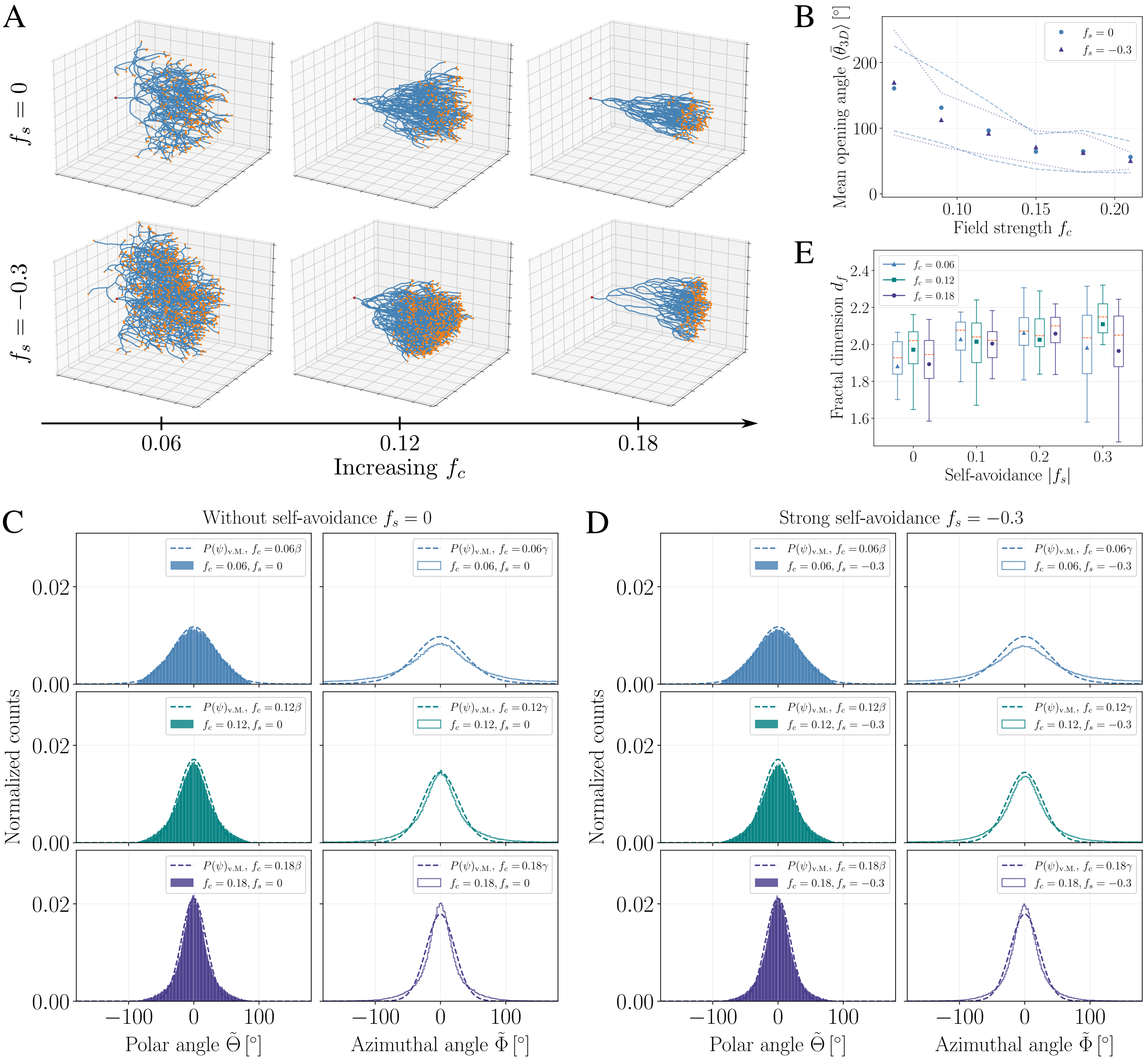

Supplementary Figure 9: **Branching morphogenesis under external guidance in a 3D geometry.** (A) Morphology diagram of BARWs in an external field (along the x-axis) obtained from simulations. Representative networks are shown for different values of the external field strength f_c both in the absence of ($f_s = 0$) and for strong ($f_s = -0.3$) self-avoidance. (B) Generalized opening angle $\bar{\theta}_{3D}$ describing a cone in 3D that contains the entire branched network decreases monotonically with increasing external field strength f_c. Self-avoidance f_s has a minimal influence on the opening angles $\bar{\theta}_{3D}$ and thus on the overall territory size of the branched networks. (C) Normalized histograms (solid bars) for the polar angles $\tilde{\Theta}$ (left) and azimuthal angles $\tilde{\Phi}$ (right) in the absence of self-avoidance ($f_s = 0$) can be well-approximated by von Mises distirbutions (dashed lines) by using the fit parameters β and γ, respectively. The angle distributions are shifted ($\tilde{\Theta} = \Theta - \pi/2$ and $\tilde{\Phi} = \Phi - \pi$) for visualization. (D) Same normalized histograms for the polar (left) and azimuthal (right) angles for strong self-avoidance ($f_s = -0.3$), showing that self-avoidance has negligible influence on these distributions. (E) Fractal dimensions obtained by the box-counting-method depend more sensitively on self-avoidance f_s than external field strength f_c, and exhibit an overall increase with increasing f_s for each choice of f_c. For each parameter choice box plots are obtained from $n = 30$ simulation runs, with mean and median values denoted respectively by the plot markers and dashed horizontal lines (orange). The boxes are drawn from the first quartile $Q1$ to the third quartile $Q3$, and whiskers indicate 1.5 interquartile range (IQR$\equiv Q3 - Q1$), i.e. max$= Q3 + 1.5\,$IQR, min$= Q1 - 1.5\,$IQR.

Fig. 9(D), indicating that branch orientations in 3D are predominantly regulated by the external field and not by local self-avoidance, in agreement with our results in 2D.

Finally, we turned to the fractal dimension of the 3D branched networks to test the effects of self-

Diffusion coefficient D	$0.031^{\dagger}$, 0.041	Mobility coefficient μ	$0.181^{\dagger}$, 0.196
Branching probability p_b	$0.03^{\dagger}$, 0.05	Maximal time $t_{\max}$	$300^{\dagger}$, 200, 150
Max. branching angle $\delta\varphi_b$ in 2D	$\pi/2$	Self-avoidance radius R_s	3ℓ, 8ℓ
Max. elongation angle $\delta\varphi_e$ in 2D	$\pi/10$	Annihilation radius R_a	1.5ℓ
Branching angle Σ_b in 3D	$U(\pi/5, \pi/2)$	Fit parameter χ for Fig. 4b (main text)	$\pi/15$
Elongation angle Σ_e in 3D	$U(0, \pi/10)$	Fit parameter α for Supplementary Fig. 7A	6
Length ℓ of branch segments	1	Fit parameters β and γ for Supplementary Figs. 9(C-D)	11, 8

Table 1: Main coefficients of the model, fit parameters, and parameter values used in the simulations. Values indicated by the dagger (†) superscript correspond to simulations of BARWs in an axial field, shown in Fig. 1 of the main text. The simulations for the radial field in the 2D system were performed until the maximal time $t_{\max} = 200$ while simulations in a 3D geometry were performed until $t_{\max} = 150$. The two self-repulsion radii $R_s = 3\ell$ and $R_s = 8\ell$ refer to simulations used in the main text and Supplementary Note 2.4.3, respectively. $U(\cdot, \cdot)$ denotes uniform distributions for the parameters Σ_b and Σ_e with the range given in the brackets.

avoidance on the space-filling features. Interestingly, even though the fractal dimensions increased with increasing self-avoidance f_s, this effect was weaker compared to the 2D case. Fractal dimensions did not change markedly for different values of the external field strength f_c, see Supplementary Fig. 9(E). We thus conclude that space-filling properties thus depend more sensitively on branching dimensionality.

2.7 Parameter values used in the simulations

In Table 1 we list the values for the diffusion D and mobility μ coefficients as predicted by our continuum model, see Eq. (S18), fitting parameters and remaining parameters used in the simulation setup.

3 Supplementary Note 3: Experimental model system and methods

3.1 Coarse-grained reconstruction of neuronal filaments

To obtain quantitative information on the branch numbers, branch lengths and probabilities of branching events, we needed to convert the manually reconstructed filament images into skeletonized datasets to extract coordinates. To achieve this, we first conducted the following analysis using custom scripts in Python: The manual reconstruction images of filament and fin borders were loaded and separated according to the channel information. After binarization, images were skeletonized with Lee's algorithm [13] implemented in scikit-image (v. 0.17.1) [14]. The Skan module (v. 0.9) [15] yielded a vector representation of filaments and border outlines. Finally, vector size and position were adjusted to correct for differences in input image resolution.

After the skeletonization with the Skan module, we still had to define hierarchical trees with a well-defined orientation for the branches. Moreover, the skeletonized networks now consisted of elementary vectors that only attained a discrete set of local angle values (integer multiples of $\pi/4$) and had a length of about a single pixel. We therefore developed an algorithm in order to coarse-grain the networks starting from an initial point of origin. The resulting networks then consisted of discrete vectors of a

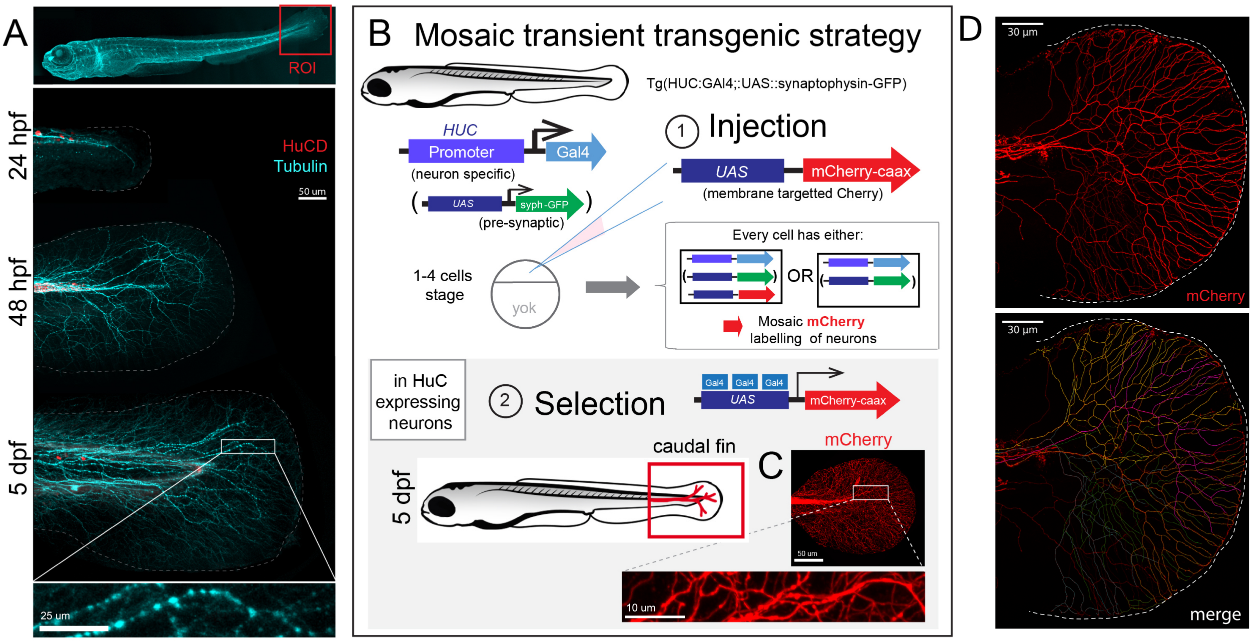

Supplementary Figure 10: **Developmental stages of caudal fin innervation, schematic of the mCherry-based experimental strategy and *in vivo* caudal fin imaging.** (A) Developmental stages of zebrafish caudal fin innervation. Top panel - 5 dpf zebrafish wholemount with red box indicating the Region of Interest (ROI): caudal fin. Several other features such as neuromasts and lateral line are visible. All tubulin positive fibers are stained with anti-acetylated tubulin antibody and presented in cyan and HuC positive cell bodies are presented in red. Different developmental stages are presented –24 hpf, 48 hpf and 5 dpf. First sensory innervation starts to appear in the caudal fin area at 24 hpf, developing into a dense network of innervation by 48 hpf (egg hatching) and reaches maximum density at 5 dpf (swimming behavior). HuC/D positive cell bodies and Tubulin positive neuronal projections are visible on all stages. Caudal fin outline is marked with the dashed lines. Magnified inset on 5 dpf stage reveals the puncta-like tubulin immunostaining which compromised potential 3D reconstruction of single neuron peripheral arborization. Indeed, a continuous signal would be preferable for reliable reconstructions, as well as sparse labelling of neurons, justifying our mCherry-based strategy. (B) Experimental scheme of our Mosaic transient transgenic strategy that lead to mCherry mosaic labelling of neurons in 5 dpf zebrafish. (1) Using GAL4-Upstream Activating Sequence (GAL4-UAS) methodology, we generated transient transgenic fish in which a sparse number of neurons will express mCherry. Injection of genetic vector carrying UAS-mCherry-caax construction to 1-4 cell stage fertilized egg of Tg(HUC:GAL4;UAS:synaptophysin-GFP) lead to mosaic UAS-mCherry caax incorporation in some cells, while in the transgenic fish line Tg(HUC:GAL4;UAS:synaptophysin-GFP) synaptophysin fused GFP is expressed at the presynaptic site in presumably all HUC expressing cells (arrowhead direction corresponds to transcription and translation processes). As a result, mCherry mosaic expression will be visible in cell membranes in occasional neurons. (2) During selection process only zebrafish with labelled neurons in the caudal fin were considered for further analysis. Selection was performed at 5 dpf to ensure developed branching of caudal fin neurons. Zebrafish with mCherry fluorescence in the caudal fin were manually collected and used for the analysis. (C) *In vivo* confocal imaging of 5 dfp mCherry-caax-XHUC:GAL4:synaptophysin-GFP zebrafish caudal fin. Magnified inset shows lack of punctiform artifacts. This feature makes our mCherry reporter line preferable for neuronal branching reconstruction and thus was used in all experiments to visualize generation of neuronal trees. (D) mCherry labelled neuronal cell membranes (top - same as Fig. 2a of the main text), vizualized together with manually reconstructed filament trees (bottom, different colors overlaid), showing the overall faithfulness of the reconstructions. Overall, $n = 8$ reconstructed neuronal filaments from $N = 4$ larvae were analyzed.

pre-defined stepsize ℓ, which had local angles with a finer distribution of values.

For the coarse-graining algorithm, we first label all branching points in the network by identifying the three-valent vertices. We then start the coarse-graining loop by defining an "active tip" which is the vector closest to the origin. This active tip will evolve by "scanning" an area of a certain radius R for the underlying skeletonized data points and taking discrete steps (of average size $\langle s \rangle$ determined mainly by the scanned radius R) according to the following rules:

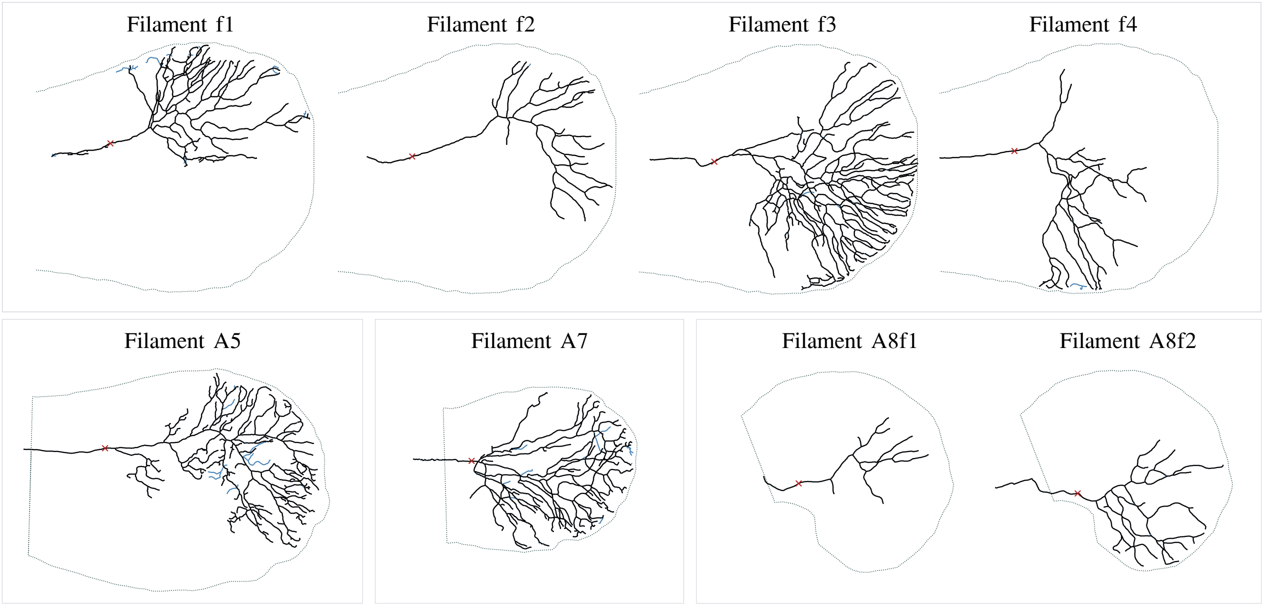

Supplementary Figure 11: **Reconstruction of experimental data.** The data points shown in black correspond to the final coarse-grained coordinates that are used in the analysis, whereas the skeletonized data from the reconstructed images are shown in blue. Filaments from the same fish are displayed in the same box. The fin edges were identified manually and are indicated by the thin dashed lines. Data acquisition corresponds to 5 dpf for all 8 samples.

(i) *Transition towards branching point*: If there is a branching point in the scanned area of radius R, the active tip will jump towards this point in the next time step.

(ii) *Elongation*: If there is no branching point in the scanned neighborhood, the active tip will try to move forward with respect to its current polarity by defining a "polarity cone" that provides a radial slice of the scanned neighborhood in the direction of the tip, and selecting the furthest data point of the underlying skeletonized network within the polarity cone in the next time step.

(iii) *Branching*: If the active tip itself is a branching point (which will necessarily be the case for a tip that undergoes the transition (i) above), it will search for two data points as progenies and produce two active tips at these coordinates in the next time step. The triangle connecting the two progenies and the active tip is required to have a minimal angle value at the vertex of the active tip in order to prevent branching events into the same branch of the underlying network.

(iv) *Annihilation*: If the above conditions are not fulfilled, the active tip will become inactive in the next time step, *i.e.*, it will not be iterated further in the loop.

In some cases, two active tips start "invading" the same branch due to noisy regions in the original raw images. In order to prevent such events, we implement the additional rule that when an active tip "moves" on an already processed data point, it will immediately terminate. In the final data processing step, we then revise the data of the corresponding branch such that it acquires the generation label and orientation of the "older" active tip that has a longer ancestral lineage.

This set of rules closely resembles the simulation setup for BARWs and provided a hierarchical tree for each sample from the experiments. After the coarse-graining loop, each processed datapoint was assigned a generation label and a local angle value determined by the vector connecting its positions at time t and in the previous time step $t - \tau$. The resulting coarse-grained coordinates for the $n = 8$ networks analyzed here are shown in Supplementary Fig. 11 in black, where the underlying skeletonized

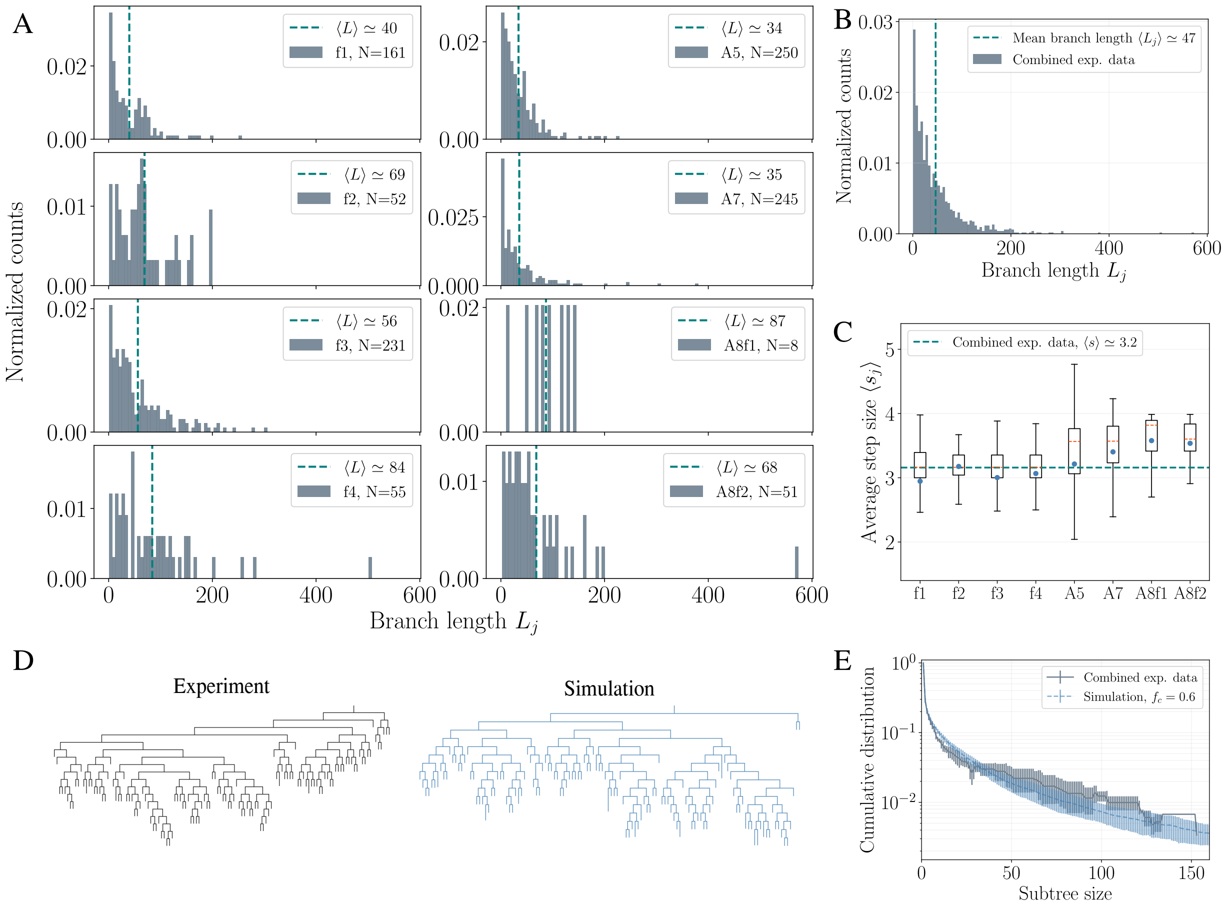

Supplementary Figure 12: **Statistics of experimental and simulated 2D trees.** (A) Normalized histograms of branch lengths (in pixels) of individual filaments after the reconstruction of experimental data with the coarse-graining algorithm. The number of branches N for each sample are indicated in the inset. Dashed vertical lines represent the mean branch length $\langle L_j \rangle$ with $j = 1, \ldots, N$ for the individual samples. (B) Normalized histogram of branch lengths for the combined data from all $n = 8$ filaments. Dashed vertical line represents the average branch length $\langle L_j \rangle$ of the combined data. (C) Individual step size distributions obtained from the coarse-graining algorithm for each sample. Box plots include $n = 2090, 1179, 4253, 1372, 2594, 2454, 359$ and 843 steps respectively for the filaments f1, f2, f3, f4, A5, A7, A8f1 and A8f2. Mean and median values are represented respectively by the plot markers (circle) and dashed horizontal lines (orange). The boxes are drawn from the first quartile $Q1$ to the third quartile $Q3$, and whiskers indicate 1.5 interquartile range (IQR$\equiv Q3 - Q1$), i.e. max$= Q3 + 1.5\,$IQR, min$= Q1 - 1.5\,$IQR. The mean branch size $\langle L_j \rangle$ (dashed line in panel B) and the average step size $\langle s \rangle$ of the combined dataset (dashed horizontal line) can be used to estimate the average number of steps in a typical branch via $\langle L_j \rangle / \langle s \rangle \simeq 14$. (D) Comparison of tree topologies between two exemplary networks obtained from simulations (right) and experiments (left), emphasizing the common stochasticity and heterogeneity in subtree sizes. (E) Cumulative subtree size distributions averaged over all branch generations exhibit large tails both in the experimental (gray) and simulation (blue) data. Lines and error bars indicate mean ± SDs from $n = 100$ simulations and $n = 8$ filaments analyzed.

networks are presented in blue. Coarse-grained coordinates of individual filaments can be found as source data in Ref. [16].

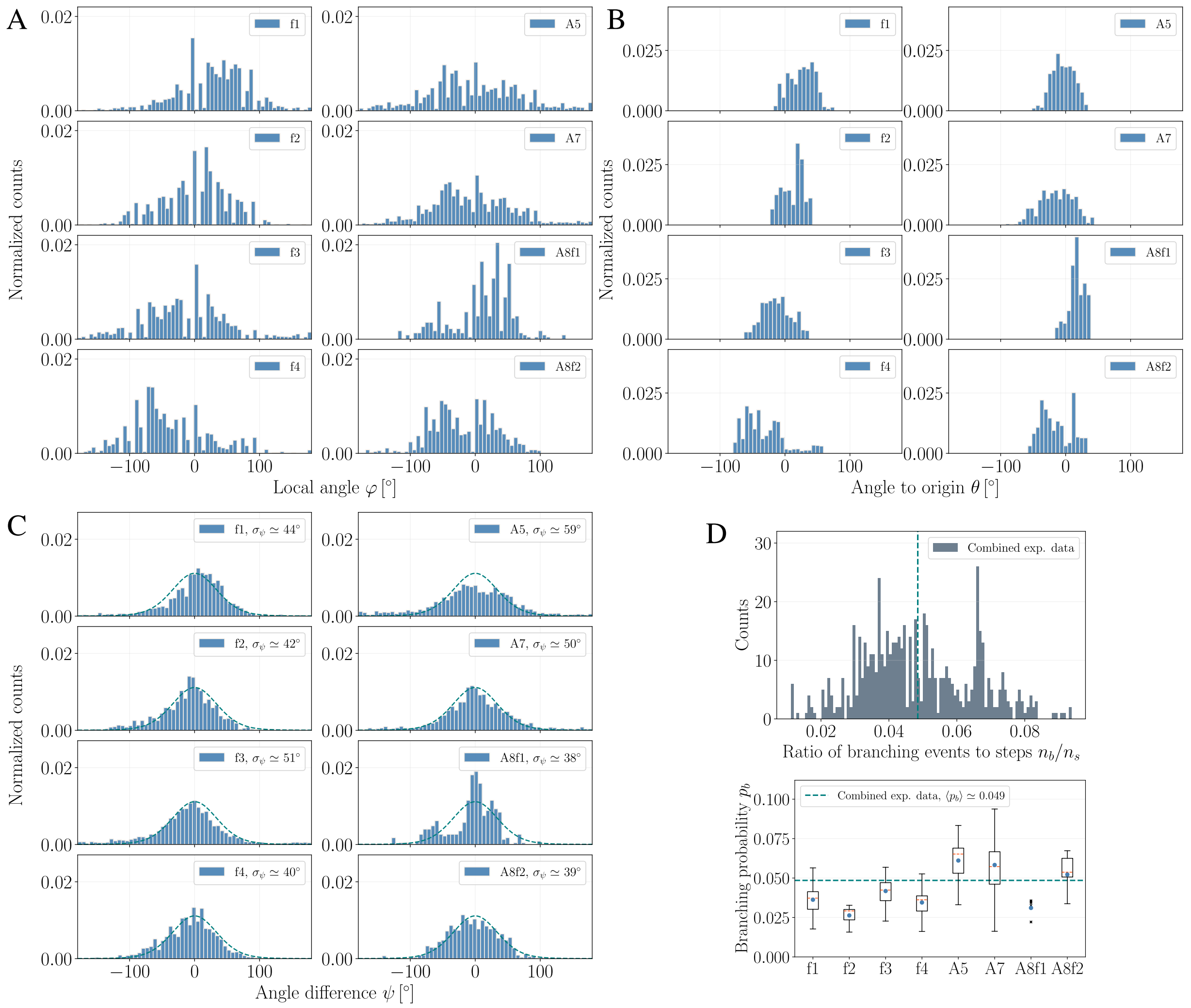

Supplementary Figure 13: **Alignment angle and branching statistics plotted for individual neurons.** (A-C) Angle distributions obtained from experimental data. Normalized histograms of the (A) local angle φ, (B) angle to origin θ, and (C) angle difference ψ values for the individual filaments. Despite significant fluctuations, the latter histograms can be well-described by the von Mises distributions (dashed lines) with an estimated branching probability of $p_b = 0.05$ and a field strength of $f_c = 0.6$. (D) Estimation of the branching probability p_b for the experimental data from the ratio of the number of branching events n_b to the number of steps n_s until the endpoint ("leaf") of each branch lineage is reached. (Top) Histogram of the ratio n_b/n_s for the combined data from $n = 8$ filaments. (Bottom) Estimates for p_b for the individual filaments. Box plots are obtained from $n = 92, 30, 126, 28, 133, 144, 8$ and 25 ratios n_b/n_s respectively for the filaments f1, f2, f3, f4, A5, A7, A8f1 and A8f2. Mean and median values are represented respectively by the plot markers (circle) and dashed horizontal lines (orange). For the filament A8f1, individual data points are displayed (crosses). The boxes are drawn from the first quartile $Q1$ to the third quartile $Q3$, and whiskers indicate 1.5 interquartile range (IQR$\equiv Q3 - Q1$), i.e. max$= Q3 + 1.5\,$IQR, min$= Q1 - 1.5\,$IQR. Average branching probability of the combined data is estimated as $\langle p_b \rangle \simeq 0.049$ (dashed horizontal line).

3.2 Analysis of experimental data

3.2.1 Distribution of branch lengths and subtree topologies

A measure that is intimately linked to the probability of branching is the average branch length $\langle L_j \rangle$ of a filament with $j = 1, \ldots, N$, where N denotes the total number of branches in the tree. For a network with a high *a priori* branching probability, one would expect to find short branches on average due to the frequent branching events. To quantify this, we estimated the branch lengths L_j by calculating

the total length $\Sigma_k s_k$ of all branch segments (discrete steps of size s_k) between the starting and end points of each branch. The normalized histograms of branch lengths for the individual filaments are displayed in Supplementary Fig. 12(A). Importantly, the combined data from $n = 8$ samples showed an exponentially decaying tail (see Fig. 2f in the main text) with an average branch length of $\langle L_j \rangle \simeq 46$, see Supplementary Fig. 12(B). This is what is expected in a stochastic branching process, validating a key assumption of the framework of BARWs. We also calculated the average step sizes corresponding to single steps of the coarse-graining algorithm described above, and obtained a mean step size of $\langle s \rangle \simeq 3.2$ from the combined dataset, see Supplementary Fig. 12(C). The mean step size can be used to determine the normalized branch lengths $\langle L_j \rangle / \langle s \rangle \simeq 14$ to compare the lengths with the simulation data (as shown in Fig. 2f in the main text).

Another signature of the BARW framework, as opposed to alternative theories of branching morphogenesis, that the subtree size structure of the networks should exhibit a strong heterogeneity with both large and small subtrees. The size of a subtree is defined by the total number of branches in it [17]. In agreement with this framework, tree topologies obtained from the experiment were quite non-stereotypical and showed a similar heterogeneity as those from simulations, see Supplementary Fig. 12(D). Furthermore, more quantitatively, comparing the cumulative distributions of subtree sizes from experiments and simulations, as averaged over all generations for each network, also showed an overall good agreement, see Supplementary Fig. 12(E). These results further supported the applicability of the BARW model to the neuronal branching system.

3.2.2 Estimation of the branching probability

A key parameter to determine for the comparison of our theoretical results with the experimental data is the branching probability p_b of the networks. One can estimate p_b from the measured distributions of branch lengths because the average branch length depends in general inversely on the branching probability, *i.e.* $\langle L_j \rangle \propto 1/p_b$. However, due to the frequent annihilation events the distributions of branch lengths in fact underestimate the average branch length, and thus lead to a high branching probability p_b that generate networks qualitatively different from the experimental observations. We therefore decided to estimate p_b by directly counting the number of branching events n_b for each lineage and taking its ratio to the total number of branch segments (steps) n_s for that lineage. The branching probability p_b of a network then corresponds to the average ratio $\langle \frac{n_b}{n_s} \rangle$ from all lineages of branches ending at a leaf. Supplementary Fig. 13(D) displays the normalized histograms for the ratio $\frac{n_b}{n_s}$ obtained from the combined dataset of $n = 8$ filaments (D1) and the estimates of p_b for the individual samples (D2). The average branching probability obtained from the combined data of $\langle p_b \rangle \simeq 0.05$ was finally used to generate the branching networks of the BARW simulations and to compare the results on angular alignment by determining the diffusion and mobility coefficients given in Eq. (S18).

3.2.3 Angle distributions and fractal dimensions for individual samples

To obtain the angle distributions we first needed to clarify the coordinate of the origin for each individual sample. Because the initial branch of some filaments was located in the spinal cord outside of the fin region, we did not take the starting point of the initial branches as the origin. Instead, an alternative choice which had the advantage of being systematic was to first locate the boundary of the fin tissue with respect to the anterior-posterior axis, and then fix a central point that has the same distance to the boundary for all samples from the same fish. After this identification, angles to origin θ and angle differences ψ could be calculated for each node in the coarse-grained networks. The normalized histograms for the local angles φ, angles to origin θ, and the angle differences ψ for the individual filaments are shown in Supplementary Fig. 13(A-C). The histograms for the local angle φ and the angle to the origin θ attained rather irregular shapes, whereas the histograms for the angle differences ψ for

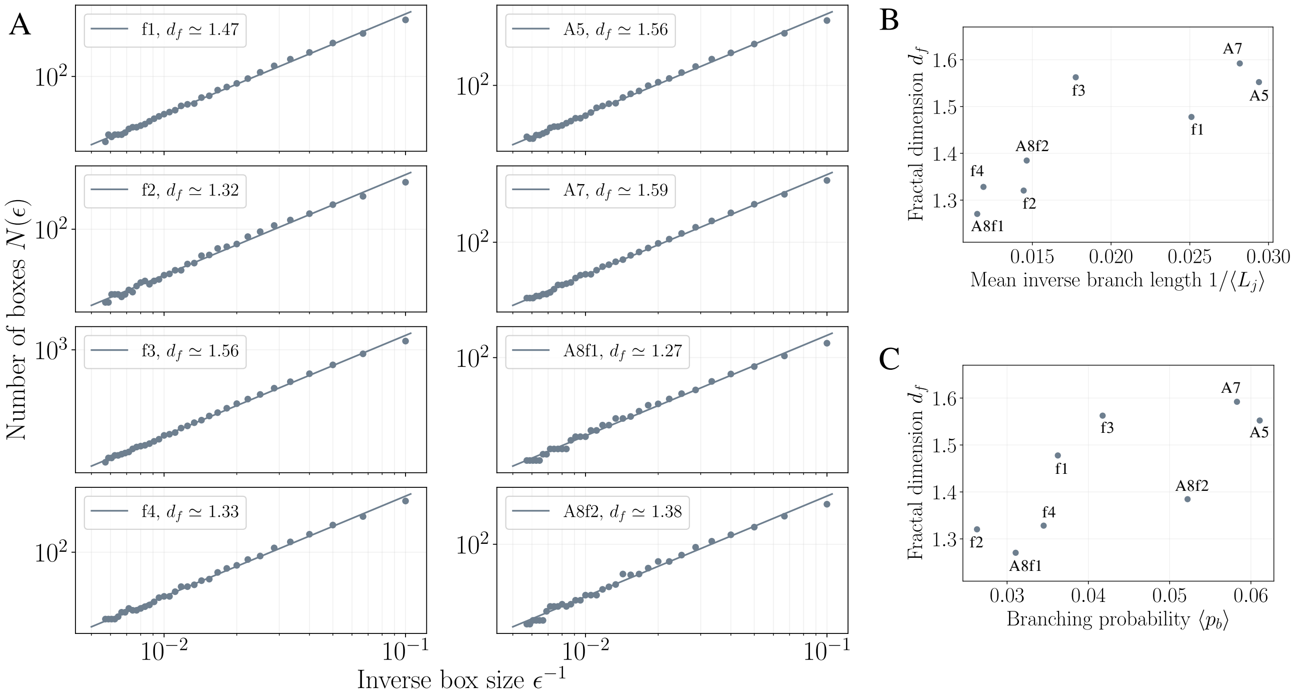

Supplementary Figure 14: **Space-filling properties plotted for each individual neuron.** (A) Fractal dimensions of the individual filaments obtained from experimental data using the box-counting method: Box sizes of varying sizes (from $\epsilon_{\min} \simeq 3\langle s \rangle$ to $\epsilon_{\max} \simeq 3\langle L_j \rangle$) are used to count the number of nonempty boxes $N(\epsilon)$ for individual networks. (B) Estimated fractal dimensions correlate with the mean inverse branch lengths $1/\langle L_j \rangle$ and (C) with the estimated branching probabilities $\langle p_b \rangle$ of the individual samples. Note that the latter values are not strictly proportional to the inverse branch lengths.

most of the individual samples could be well-approximated by the analytical predictions (Supplementary Fig. 13(C), dashed lines). Importantly, the prediction held for both denser and sparser networks, which is a key prediction of the model, and wouldn't hold if for instance the directionality was emerging from the repulsion between a dense network of tip/branches (see Supplementary Figs. 3(D)).

Finally, in Supplementary Fig. 14 we display the fractal dimensions for the individual samples obtained by the box-counting method. For this purpose, we selected box sizes gradually increasing from $\epsilon_{\min} \simeq 3\langle s \rangle$ to $\epsilon_{\max} \simeq 3\langle L_j \rangle$ to describe a well-defined scaling behavior (*i.e.* to omit boundary effects arising from too large or too small boxes). The fractal dimensions showed a rather robust correlation with the inverse of the mean branch lengths and with the branching probability, even though these two measures were not strictly proportional for all samples, see Supplementary Figs. 14(B) and (C).

References

[1] Edward A Codling, Michael J Plank, and Simon Benhamou. Random walk models in biology. *Journal of the Royal society interface*, 5(25):813–834, 2008.

[2] NA Hill and D-P Häder. A biased random walk model for the trajectories of swimming microorganisms. *Journal of theoretical biology*, 186(4):503–526, 1997.

[3] M Amdaoud, M Vallade, C Weiss-Schaber, and Irina Mihalcescu. Cyanobacterial clock, a stable phase oscillator with negligible intercellular coupling. *Proceedings of the National Academy of Sciences*, 104(17):7051–7056, 2007.

[4] Hannes Risken. Fokker-planck equation. In *The Fokker-Planck Equation*. Springer, 1996.

[5] Ralf Metzler and Joseph Klafter. The random walk's guide to anomalous diffusion: a fractional dynamics approach. *Physics reports*, 339(1):1–77, 2000.

[6] R Metzler. Non-homogeneous random walks, generalised master equations, fractional fokker-planck equations, and the generalised kramers-moyal expansion. *The European Physical Journal B-Condensed Matter and Complex Systems*, 19(2):249–258, 2001.

[7] Kanti V Mardia and Peter E Jupp. *Directional statistics*, volume 494. John Wiley & Sons, 2009.

[8] Edouard Hannezo, Colinda LGJ Scheele, Mohammad Moad, Nicholas Drogo, Rakesh Heer, Rosemary V Sampogna, Jacco Van Rheenen, and Benjamin D Simons. A unifying theory of branching morphogenesis. *Cell*, 171(1):242–255, 2017.

[9] Vanessa Lanoue and Helen M Cooper. Branching mechanisms shaping dendrite architecture. *Developmental Biology*, 451(1):16–24, 2019.

[10] Jeremy N Kay, Monica W Chu, and Joshua R Sanes. Megf10 and megf11 mediate homotypic interactions required for mosaic spacing of retinal neurons. *Nature*, 483(7390):465–469, 2012.

[11] S Lawrence Zipursky and Wesley B Grueber. The molecular basis of self-avoidance. *Annual review of neuroscience*, 36:547–568, 2013.

[12] Amrutha Palavalli, Nicolás Tizón-Escamilla, Jean-François Rupprecht, and Thomas Lecuit. Deterministic and stochastic rules of branching govern dendrite morphogenesis of sensory neurons. *Current Biology*, 31(3):459–472, 2021.

[13] Gilles Bertrand and Grégoire Malandain. A note on" building skeleton models via 3-d medial surface/axis thinning algorithms". *Graphical Models and Image Processing*, 57(6):537–538, 1995.

[14] Stefan Van der Walt, Johannes L Schönberger, Juan Nunez-Iglesias, François Boulogne, Joshua D Warner, Neil Yager, Emmanuelle Gouillart, and Tony Yu. scikit-image: image processing in python. *PeerJ*, 2:e453, 2014.

[15] Juan Nunez-Iglesias, Adam J Blanch, Oliver Looker, Matthew W Dixon, and Leann Tilley. A new python library to analyse skeleton images confirms malaria parasite remodelling of the red blood cell membrane skeleton. *PeerJ*, 6:e4312, 2018.

[16] Mehmet Can Ucar. Source data for the manuscript "Theory of branching morphogenesis by local interactions and global guidance" [Data set]. Zenodo. https://doi.org/10.5281/zenodo.5257161, August 2021.

[17] Colinda LGJ Scheele, Edouard Hannezo, Mauro J Muraro, Anoek Zomer, Nathalia SM Langedijk, Alexander Van Oudenaarden, Benjamin D Simons, and Jacco Van Rheenen. Identity and dynamics of mammary stem cells during branching morphogenesis. *Nature*, 542(7641):313–317, 2017.
