# Supplementary material for: Theory of branching morphogenesis by local interactions and global guidance
Source: Nat Commun. 2021 Nov 24;12:6830. doi: 10.1038/s41467-021-27135-5 (PMC8613190; doi:10.1038/s41467-021-27135-5)
Supplement: Supplementary file 3 — Description of Additional Supplementary Files [file 41467_2021_27135_MOESM3_ESM.pdf]

Description of Additional Supplementary Files

File Name: Supplementary Movie 1
Description: Different manually reconstructed neuronal trees of the zebrafish caudal fin.

File Name: Supplementary Movie 2
Description: Exemplary simulation of a BARW in a three-dimensional geometry with external guidance along the vertical axis.
