# Supplementary material for: Theory of branching morphogenesis by local interactions and global guidance
Source: Nat Commun. 2021 Nov 24;12:6830. doi: 10.1038/s41467-021-27135-5 (PMC8613190; doi:10.1038/s41467-021-27135-5)
Supplement: Supplementary file 6 — Reporting Summary [file 41467_2021_27135_MOESM6_ESM.pdf]

nature portfolio

Corresponding author(s): Hannezo, Hadjab

Last updated by author(s): 2021/10/28

Reporting Summary

Nature Portfolio wishes to improve the reproducibility of the work that we publish. This form provides structure for consistency and transparency in reporting. For further information on Nature Portfolio policies, see our Editorial Policies and the Editorial Policy Checklist.

Statistics

For all statistical analyses, confirm that the following items are present in the figure legend, table legend, main text, or Methods section.

n/a	Confirmed	
☐	☒	The exact sample size (*n*) for each experimental group/condition, given as a discrete number and unit of measurement
☐	☒	A statement on whether measurements were taken from distinct samples or whether the same sample was measured repeatedly
☒	☐	The statistical test(s) used AND whether they are one- or two-sided<br>*Only common tests should be described solely by name; describe more complex techniques in the Methods section.*
☒	☐	A description of all covariates tested
☐	☒	A description of any assumptions or corrections, such as tests of normality and adjustment for multiple comparisons
☐	☒	A full description of the statistical parameters including central tendency (e.g. means) or other basic estimates (e.g. regression coefficient) AND variation (e.g. standard deviation) or associated estimates of uncertainty (e.g. confidence intervals)
☒	☐	For null hypothesis testing, the test statistic (e.g. *F*, *t*, *r*) with confidence intervals, effect sizes, degrees of freedom and *P* value noted<br>*Give P values as exact values whenever suitable.*
☒	☐	For Bayesian analysis, information on the choice of priors and Markov chain Monte Carlo settings
☒	☐	For hierarchical and complex designs, identification of the appropriate level for tests and full reporting of outcomes
☒	☐	Estimates of effect sizes (e.g. Cohen's *d*, Pearson's *r*), indicating how they were calculated

Our web collection on statistics for biologists contains articles on many of the points above.

Software and code

Policy information about availability of computer code

Data collection	Zeiss Zen Blue software (version 3.3) for confocal microscope LSM 800
Data analysis	Imaris 8.0, ImageJ 2.1.0, custom Python code are available on the following doi link: 10.5281/zenodo.5257161

For manuscripts utilizing custom algorithms or software that are central to the research but not yet described in published literature, software must be made available to editors and reviewers. We strongly encourage code deposition in a community repository (e.g. GitHub). See the Nature Portfolio guidelines for submitting code & software for further information.

Data

Policy information about availability of data

All manuscripts must include a data availability statement. This statement should provide the following information, where applicable:

- Accession codes, unique identifiers, or web links for publicly available datasets
- A description of any restrictions on data availability
- For clinical datasets or third party data, please ensure that the statement adheres to our policy

Data is available on the following doi link: 10.5281/zenodo.5257161

Field-specific reporting

Life sciences study design

All studies must disclose on these points even when the disclosure is negative.

Sample size	No sample size calculation was performed. Sample size was chosen to n=8 neurons from N=4 different larvae to ensure statistical power and be able to check for variability between different embryos.
Data exclusions	No data was excluded from the analysis.
Replication	Reproducibility was verified by performing all measurements in 3 replicas with multiple individual samples in each group. All replications were successful.
Randomization	No randomization was performed as we were only considering a WT condition, so that no comparisons without another condition (e.g. mutant, which could have been randomized was performed. Quantification methods were applied equally to all conditions and experiments.
Blinding	Blinding was not relevant to our study as we were only considering a WT condition, so that no comparisons without another condition (e.g. mutant, which could have been blinded) was performed. Quantification methods were applied equally to all conditions and experiments.

Reporting for specific materials, systems and methods

We require information from authors about some types of materials, experimental systems and methods used in many studies. Here, indicate whether each material, system or method listed is relevant to your study. If you are not sure if a list item applies to your research, read the appropriate section before selecting a response.

Materials & experimental systems

n/a	Involved in the study	
☐	☒	Antibodies
☒	☐	Eukaryotic cell lines
☒	☐	Palaeontology and archaeology
☐	☒	Animals and other organisms
☒	☐	Human research participants
☒	☐	Clinical data
☒	☐	Dual use research of concern

Methods

n/a	Involved in the study	
☒	☐	ChIP-seq
☒	☐	Flow cytometry
☒	☐	MRI-based neuroimaging

Antibodies

Antibodies used

Tubulin (Acetyl Lys40) antibody [6-11B-1] Cat No. GTX16292
Anti-HuC/HuD protein antibody. Cat. No. ab210554
Goat anti-Rabbit IgG (H+L) Cross-Adsorbed Secondary Antibody, Alexa Fluor 555 Catalog # A-21429
Goat anti-mouse IgG (H+L) Cross-Adsorbed Secondary Antibody, Alexa Fluor 647 Catalog # A-21235
Alexa Fluor 555 (Product # A-21429) was performed using HepG2 cells stained with alpha-1 antitrypsin Rabbit Polyclonal Primary Antibody (Product # PA5-16661).

Validation

All antibodies were validated by manufacturer:

Tubulin (Acetyl Lys40) antibody [6-11B-1] Cat No. GTX16292
Validation on the manufacturer website:
Picture 1 description
GTX16292 ICC/IFImage ICC/IF analysis of HeLacells using GTX16292 Tubulin (Acetyl Lys40)antibody[6-11B-1]at 1:2000 (red) with DAPI (blue). Cells were fixed and permeabilized with 4%paraformaldehyde followed by 0.5%Triton™X-100.
Picture 2 description
GTX16292 WB Image WB analysis of (1)HeLa(2) U87 (3) COS7 (4) P19 (5) rat2 (6) CHO (7) MDBK (8) MDCK cells using GTX16292 Tubulin (Acetyl Lys40)antibody[6-11B-1]at 1:2,000.

Anti-HuC/HuD protein antibody. Cat. No. ab210554
Validation on the manufacturer website:
Positive control
IHC-Wm: 2 days-post-fertilization zebrafish embryo, 24 hours-post-fertilization zebrafish embryo. WM images are provided on the manufacturer website.

Goat anti-Rabbit IgG (H+L) Cross-Adsorbed Secondary Antibody, Alexa Fluor 555 Catalog # A-21429
Goat anti-mouse IgG (H+L) Cross-Adsorbed Secondary Antibody, Alexa Fluor 647 Catalog # A-21235

Antibody testing data provided on the manufacturer website.

Rabbit IgG (H+L) Highly Cross-Adsorbed Secondary Antibody (A-21429) in ICC/IF

Immunofluorescence analysis of Goat anti-Rabbit IgG (H+L) Secondary Antibody, Alexa Fluor 555 (Product # A-21429) was performed using HepG2 cells stained with alpha-1 antitrypsin Rabbit Polyclonal Primary Antibody (Product # PA5-16661).

Mouse IgG (H+L) Cross-Adsorbed Secondary Antibody (A-21235) in ICC/IF

Immunofluorescence analysis of Goat anti-Mouse IgG (H+L) Cross-Adsorbed Secondary Antibody, Alexa Fluor® 647 conjugate was performed using HeLa cells stained with alpha Tubulin (236-10501) Mouse Monoclonal Antibody (Product # A11126).

Animals and other organisms

Policy information about studies involving animals; ARRIVE guidelines recommended for reporting animal research

Laboratory animals	Zebrafish Danio rerio, up to 5 dpf, males and females
Wild animals	The study did not involve wild animals
Field-collected samples	No samples were collected in the field
Ethics oversight	Zebrafish were raised and housed in the Karolinska Institutet core facility following established and approved procedures. The study was performed in accordance with local guidelines and regulations and approved by "Stockholms djurforsoksetiska namnd". No special ethical permit required for the experiments on Zebrafish up to 5 dpf.

Note that full information on the approval of the study protocol must also be provided in the manuscript.
